# Chromosomal Manipulation by Site-Specific Recombinases and Fluorescent Protein-Based Vectors

**DOI:** 10.1371/journal.pone.0009846

**Published:** 2010-03-24

**Authors:** Munehiro Uemura, Youko Niwa, Naoki Kakazu, Noritaka Adachi, Kazuo Kinoshita

**Affiliations:** 1 Evolutionary Medicine, Shiga Medical Center Research Institute, Moriyama, Japan; 2 Department of Environmental and Preventive Medicine, Shimane University School of Medicine, Izumo, Japan; 3 Graduate School of Nanobioscience, Yokohama City University, Yokohama, Japan; Oregon State University, United States of America

## Abstract

Feasibility of chromosomal manipulation in mammalian cells was first reported 15 years ago. Although this technique is useful for precise understanding of gene regulation in the chromosomal context, a limited number of laboratories have used it in actual practice because of associated technical difficulties. To overcome the practical hurdles, we developed a Cre-mediated chromosomal recombination system using fluorescent proteins and various site-specific recombinases. These techniques enabled quick construction of targeting vectors, easy identification of chromosome-rearranged cells, and rearrangement leaving minimum artificial elements at junctions. Applying this system to a human cell line, we successfully recapitulated two types of pathogenic chromosomal translocations in human diseases: *MYC/IgH* and *BCR/ABL1*. By inducing recombination between two *loxP* sites targeted into the same chromosome, we could mark cells harboring deletion or duplication of the inter-*loxP* segments with different colors of fluorescence. In addition, we demonstrated that the intrachromosomal recombination frequency is inversely proportional to the distance between two recombination sites, implicating a future application of this frequency as a proximity sensor. Our method of chromosomal manipulation can be employed for particular cell types in which gene targeting is possible (e.g. embryonic stem cells). Experimental use of this system would open up new horizons in genome biology, including the establishment of cellular and animal models of diseases caused by translocations and copy-number variations.

## Introduction

Modern genetic engineering depends on DNA-modifying enzymes including restriction endonucleases, ligases and polymerases. This technology has been applied to manipulation of purified DNA less than a few hundred kilobases. Broadening the range of manipulatable DNA to megabase scale would be fundamental to deepen the understanding of gene regulation in the chromosomal context. To this purpose, chromosomal manipulation in mammalian cells by Cre recombinase (a site-specific recombinase derived from bacteriophage P1, catalyzing DNA recombination between two 34-bp *loxP* sequences) after targeted integration of two *loxP* sites into defined chromosomal loci has been reported previously [1]–[8]. These studies relied exclusively on the selection of cells expressing hypoxanthine phosphoribosyltransferase (HPRT) as an indicator of recombination, and therefore, the use of HPRT-deficient cells was a prerequisite. In this study, we introduced two improvements to this technology. First, we utilized fluorescent proteins as rearrangement markers to broaden the range of cells this technology can be applied to. Second, to facilitate the otherwise cumbersome construction of targeting vectors, we adopted Gateway cloning system, which utilizes *in vitro* site-specific recombination by λ-phage-derived integrase complexes (BP and LR Clonases) [9]. Here, we demonstrate three kinds of application of our novel chromosomal manipulation: recapitulation of pathogenic chromosomal translocation, induction of copy-number variation, and assessment of proximity between gene loci.

## Results and Discussion

### New vectors for chromosomal manipulation

**Figure 1:**
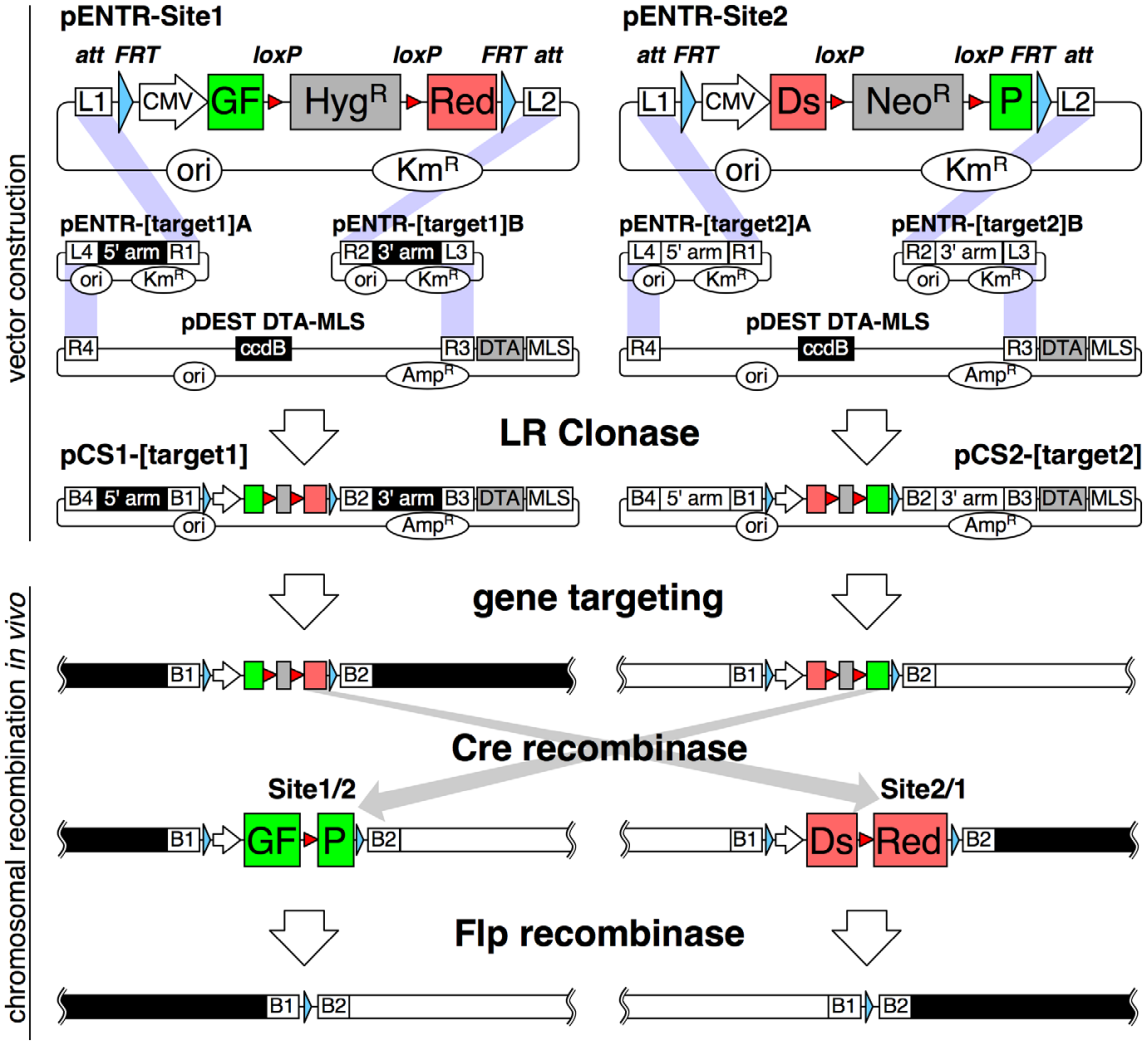
Scheme for vector construction and chromosomal recombination. (upper) pENTR-Site1 plasmid contains cytomegalovirus (CMV) promoter-driven fusion gene, the first half consisting of GFP (green box) and the last half of dimer2 (a DsRed variant, red box) with an intervening hygromycin-resistance gene (grey box) flanked by *loxP* sites (red triangles), outer two *FRT* sites (blue triangles) and *attL1* and *attL2* sites (open box). pENTR-Site2 expresses a fusion gene complementary to pENTR-Site1 harboring a neomycin-resistance gene (grey box). These vectors in addition to 5′- and 3′-targeting homology arm vectors and the pDEST DTA-MLS destination vector were assembled into targeting vectors by LR Clonase. Purple shadows connect *att* sites to be recombined. (lower) After gene targeting into homologous chromosomal regions, Cre first removes drug-resistance genes and then recombines distant *loxP* sites. After recombination, GFP and dimer2 mRNAs are spliced and expressed. Expression of Flp excises fluorescent protein genes to achieve *clean* rearrangement, leaving a 103-bp element. DTA, diphtheria toxin A; MLS, multiple linearization sites (PmeI, AscI, I-SceI, SwaI, PacI); ori, replication origin; Km^R^, kanamycin-resistance gene; Amp^R^, ampicillin-resistance gene; ccdB, bacterial *ccdB* gene.

To monitor the integrity and fate of two junctions, we generated *loxP* site-containing targeting vectors to encode green and red fluorescent proteins after recombination. As shown in Fig. 1, coding sequences of enhanced green fluorescent protein (GFP) and the red fluorescent protein variant dimer2 [10] (DsRed) were split in the middle and fused to each other with intervening drug selection markers (hygromycin- or neomycin-resistance gene) franked by *loxP* sites. Similar strategy utilizing restoration of split-fluorescent protein by Cre recombinase was previously adopted for analysis of neuronal differentiation in mice [11]. Our gene cassettes for split-fluorescent protein are bound by *FRT* and *att* (*L1* or *L2*) sites for later recognition by Flp recombinase (a site-specific recombinase derived from the 2 µm plasmid of budding yeast) and Gateway LR Clonase, respectively. These constructed vectors can be easily converted to the final gene-targeting vectors in a reaction with two homology-arm vectors and a destination vector (i.e. pDEST DTA-MLS [12]), catalyzed by LR Clonase.

In this study, we used Nalm-6, a human pre-B acute lymphoblastic leukemia cell line with high gene-targeting efficiency [13]. Before gene targeting, we stably transfected a vector expressing 4-hydroxytamoxifen (OHT)-regulated Cre recombinase (MerCreMer [14]) to Nalm-6 to obtain NCR1 cells. MerCreMer is a modified Cre both amino- and carboxyl-terminally fused with hormone-binding domain of human estrogen receptor α with mutations that reduces response to native estrogens but not its synthetic analogue OHT (Mer stands for mutated estrogen receptor). MerCreMer lacks Cre activity in the absence of OHT but is activated promptly by OHT.

After linearization at multiple linearization sites (MLS), a Site1-targeting vector was electroporated into NCR1 cells, followed by PCR screening and Southern blotting. A targeted clone was further subjected to a second targeting with a Site2 vector. Thus, double-targeted clones were obtained. Addition of OHT at a later stage can induce excision of drug-resistant markers, leaving a *loxP* site at the GFP–DsRed junction. Then, continued Cre activity would lead to recombination between the *loxP* sites in Site1 and Site2. To remove GFP and DsRed expression units and *loxP* sites, Flp recombinase was transiently expressed in GFP- and DsRed-positive cells, leaving only the 103-bp artificial sequence of *attB1*-*FRT*-*attB2* (Fig. 1). We introduced this Flp recombinase-mediated excision of fluorescent protein expression elements because presence of those promoters may perturb expression of nearby genes. In this paper, we refer to a transgenic state after Flp-mediated excision of restored fluorescent protein genes as *clean* (e.g. *clean* translocation).

### Induction of chromosomal translocation

**Figure 2:**
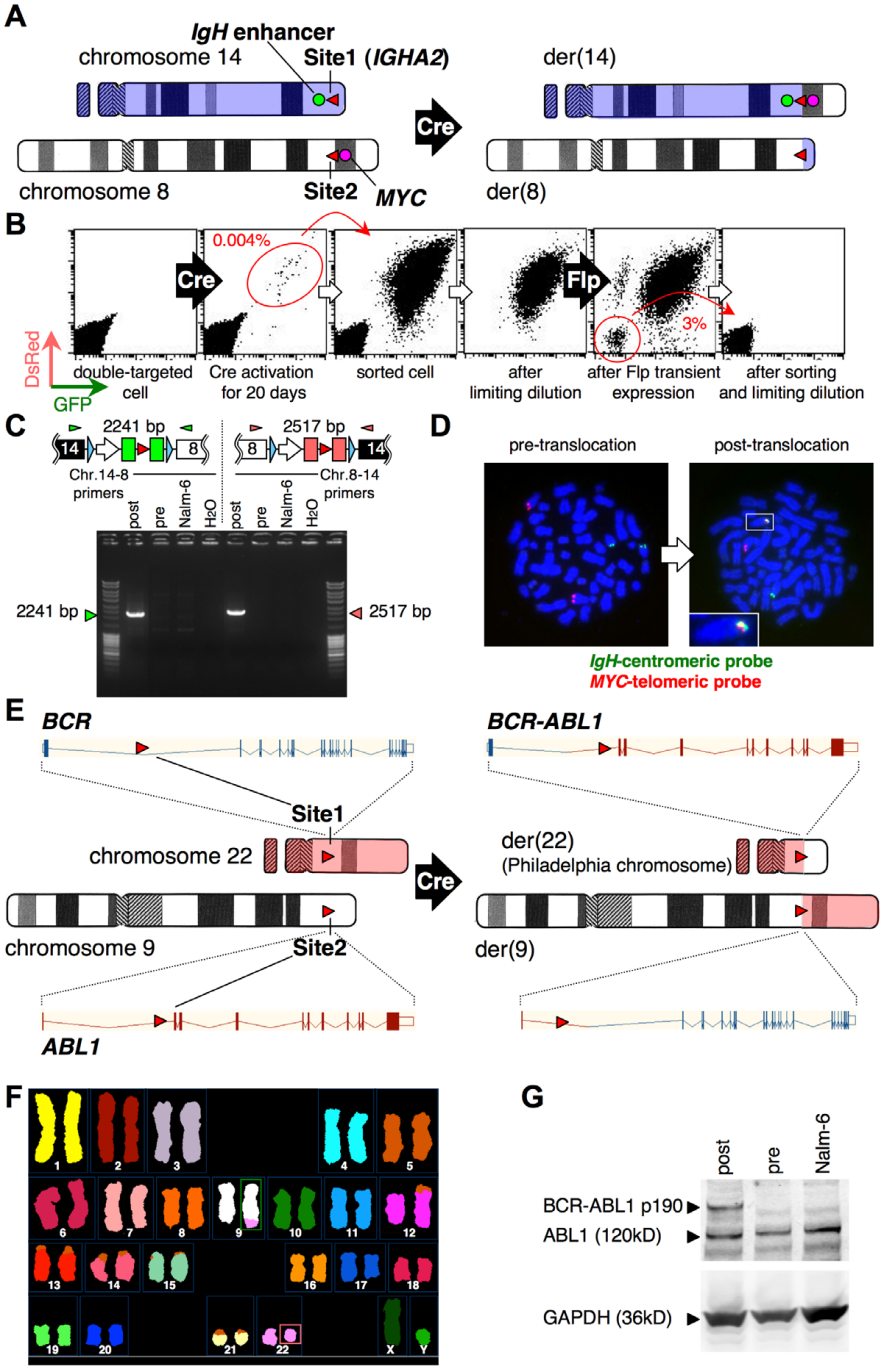
Recapitulation of *MYC/IgH* and *BCR/ABL1* translocations. A, Site1 vector (red triangle on chromosome 14) was targeted to the *IGHA2* locus of Nalm-6 cells expressing tamoxifen-regulated Cre. Site2 vector (red triangle on chromosome 8) was subsequently targeted to the *MYC* locus (red circle). The *IgH* enhancer is indicated by a green circle. B, Flow cytometric profiles of the indicated stages. Red circles indicate the sorted cell populations. C, Confirmation of translocation by PCR. PCR products of expected sizes were observed for cells after translocation (post) but not for cells before Cre expression (pre). H_2_O lanes show the no-template controls. Triangles above the transgene schemes represent primer positions (IgA2-B41-F1 and MYC-B23-R1 for green, and MYC-B41-F1 and IgA2-B23-R1 for red), respectively. D, FISH analysis demonstrating *MYC/IgH* translocation for cells after recombination by Flp, using BAC probes. Green probe hybridizes to a chromosome 14 region near Site1-integrated *IgH* locus on the centromeric side. Red probe hybridizes to a chromosome 8 region near Site2-integrated *MYC* locus on the telomeric side. These probes co-localize only after Cre-mediated translocation and appear as a yellow signal (right panel, box), magnification of which is shown in the inset. E, Site1 and Site2 vectors containing *loxP* sites (red rectangle) were targeted into the first intron of the *BCR* gene and the *ABL1* gene, respectively. Vertical bars and boxes connected with v-shaped lines indicate exon–intron structure of genes. The derivative chromosome 22 [der(22)] recapitulates the Philadelphia (Ph) chromosome. F, SKY analysis of cells with artificial *BCR/ABL1* translocation. The derivative chromosome 9 [der(9)] in the green box contains the material (pink) translocated from chromosome 22. The amount of chromosome 9 material that was translocated to chromosome 22 was too small to be resolved by this SKY analysis (Ph chromosome, pink box). The previously reported translocation between chromosomes 5 and 12 in Nalm-6 cells [23] could be also detected. G, Western blot confirming expression of the BCR-ABL1 fusion protein (190 kD) after *clean* translocation (post) with constitutive expression of ABL1 and GAPDH proteins.

To test the feasibility of this system, we recapitulated two pathogenic chromosomal translocations in human hematological malignancies: t(8;14)(q24;q32) (*MYC/IgH*) found in Burkitt lymphoma and t(9;22)(q34;q11.2) (*BCR/ABL1*) in chronic myeloid leukemia. As shown in Fig. 2A, we introduced Site1 within the immunoglobulin heavy chain α2 (*IGHA2*) locus on the telomeric side of the *IgH* 3′ enhancer in MerCreMer-expressing NCR1 cells (Fig. S1). One of the six clones with correct targeting was designated NSB1. Subsequently, the *MYC* locus of NSB1 cells was targeted by Site2 vector and four targeted clones were obtained (NSMyc, Fig. S2), of which three clones were cultured in the presence or absence of OHT for 7 days and analyzed by flow cytometry.

The frequency of GFP-DsRed double-positive cells was (1.5±1.2)×10^−5^ [mean ± standard deviation, *n* = 3]. Enforcement of Cre activity by a retroviral vector increased the frequency of double-positive cells by more than 40 fold (6.8×10^−4^), indicating that Cre activity is a major factor influencing translocation efficiency. We continued Cre activation of one clone, NSMyc23, until day 20, when we sorted the double-positive cells (Fig. 2B). The sorted cells were then subjected to limiting dilution to obtain pure clones of double-positive cells, followed by electroporation of a Flp-expression plasmid to achieve a *clean* translocation. The resulting double-negative cells were cloned by sorting and limiting dilution. Chromosomal translocation was verified by polymerase chain reaction (PCR) using *MYC/IgH* translocation-specific primers (Fig. 2C) and by fluorescence *in situ* hybridization (FISH) using bacterial artificial chromosome (BAC) probes specific to the *IgH* or the *MYC* locus (Fig. 2D).

We similarly induced *BCR/ABL1* translocation by Site1 targeting to the *BCR* locus of NCR1 cells and subsequent Site2 targeting to the *ABL1* locus (Fig. 2E, Fig. S3 and Fig. S4). The frequency of double-positive cells on day 7 of OHT stimulation was (5.9±1.8)×10^−5^ (*n* = 10). To verify production of the *BCR-ABL1* translocation, several cell clones with a *clean* translocation were obtained after transient expression of Flp recombinase in the fluorescence-positive cells followed by flow-cytometric sorting and limiting dilution for fluorescence-negative cells. Successful translocation in thus obtained cell clones was verified by FISH analysis (not shown) and spectral karyotyping (SKY, a FISH-based cytogenetic technique that allows the simultaneous identification of all 24 human chromosomes with whole chromosome painting probes labeled with different combination of fluorescent dyes) (Fig. 2F). Production of BCR-ABL1 fusion protein was confirmed by western blot analysis (Fig. 2G). Expected splicing between juxtaposed exons was confirmed by sequencing reverse-transcription PCR product (data not shown).

### Induction of copy number variation

**Figure 3:**
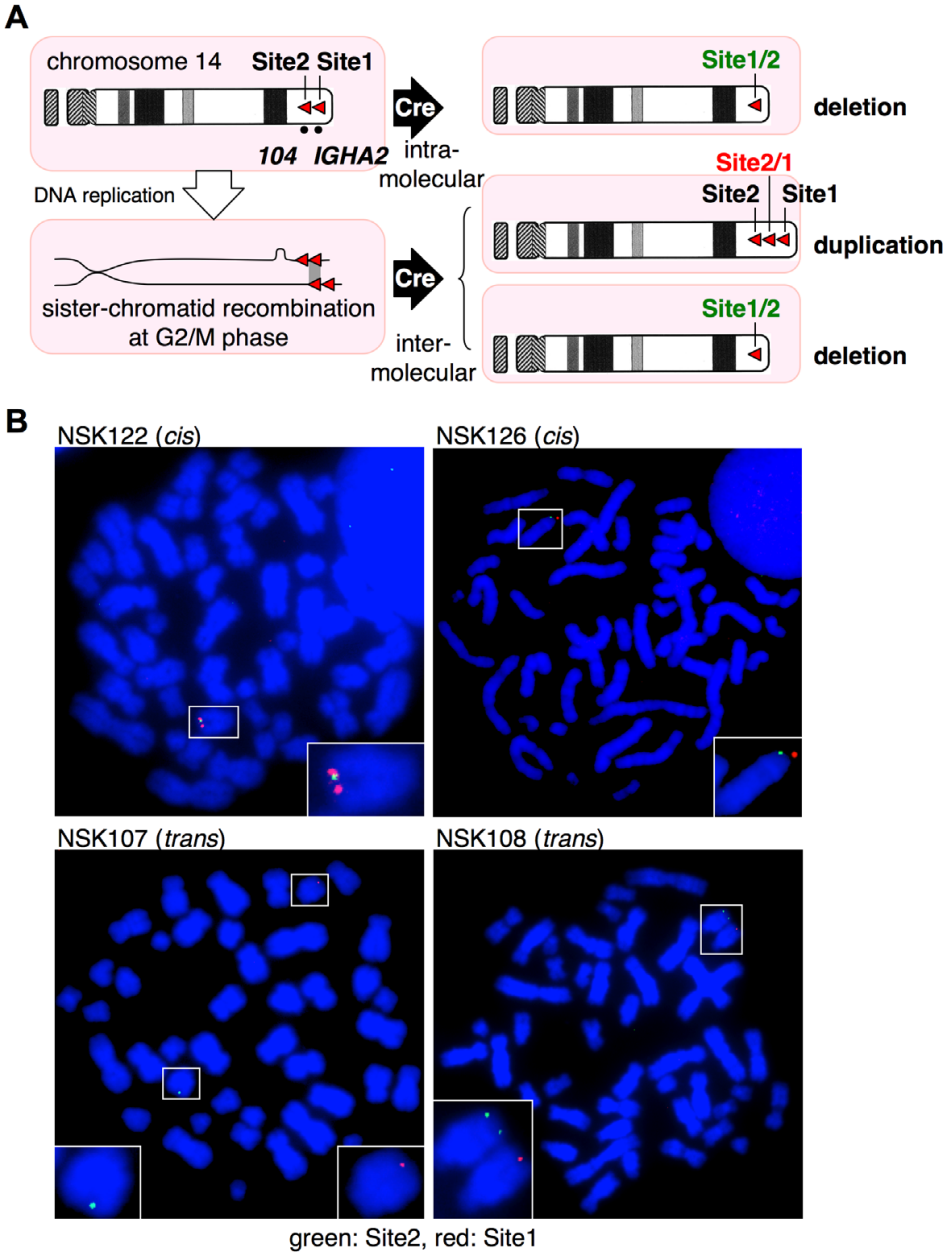
Site1 and Site2 targeting for copy-number variation. A, Possible outcome of Cre-mediated recombination between two *loxP* sites with an identical orientation in the same chromosome 14. Site1 was targeted to the *IGHA2* locus within the *IgH* gene cluster. Site2 was targeted to a region tentatively designated *104*, 1.1 Mb centromeric to the *IGHA2* loci. B, Results of representative two *cis*-targeted (NSK122 and NSK126) and two *trans*-targeted clones (NSK107 and NSK108) are shown. The chromosomes 14 with Site1 and Site2 integration harbor red and green signals, respectively, and are highlighted with boxes, magnifications of which are shown in insets.

Recently, much attention has been paid to the copy-number variation (CNV) in the human genome, which is considered to have a greater impact on the phenotype than single nucleotide polymorphism. Utilities of site-specific recombinases to generate CNV were previously reported in several organisms including yeast [15], plant [16], fly [17] and mouse [2]. To demonstrate the feasibility of our system for generating CNV, we inserted Site1 and Site2 into the same chromosome 14 (Fig. 3A) with an identical orientation. For Site2 targeting, NSB1 cells were used, which were used for recapitulation of *MYC/IgH* translocation (Fig. 2A–D) and harbor Site1 at *IGHA2* locus. Site 2 was targeted to an intergenic region between *LOC122631* and *C14orf180*, which lies 1.1-Mb apart from Site1 to the centromere. Cre activation was expected to result in two different outcomes. Recombination in the same DNA molecule leads to deletion of the inter-*loxP* segment. This causes GFP expression from fused Site1/2 (5′ half of Site1 and 3′ half of Site2) in the chromosome and transient expression of DsRed from Site2/1 (5′ half of Site2 and 3′ half of Site1) in the excised circular product that eventually disappears. Alternatively, nonallelic recombination at the G2/M phase of the cell cycle between sister chromatids leads to inter-*loxP* duplication on one chromatid and its deletion on the other (Fig. 3A). Subsequent cell division generates both cells with duplication expressing only DsRed and cells with deletion expressing only GFP. Therefore, irrespective of the two recombination modes (intramolecular and intermolecular recombination), GFP- and DsRed-positive cells represent cells with deletion and duplication, respectively.

**Figure 4:**
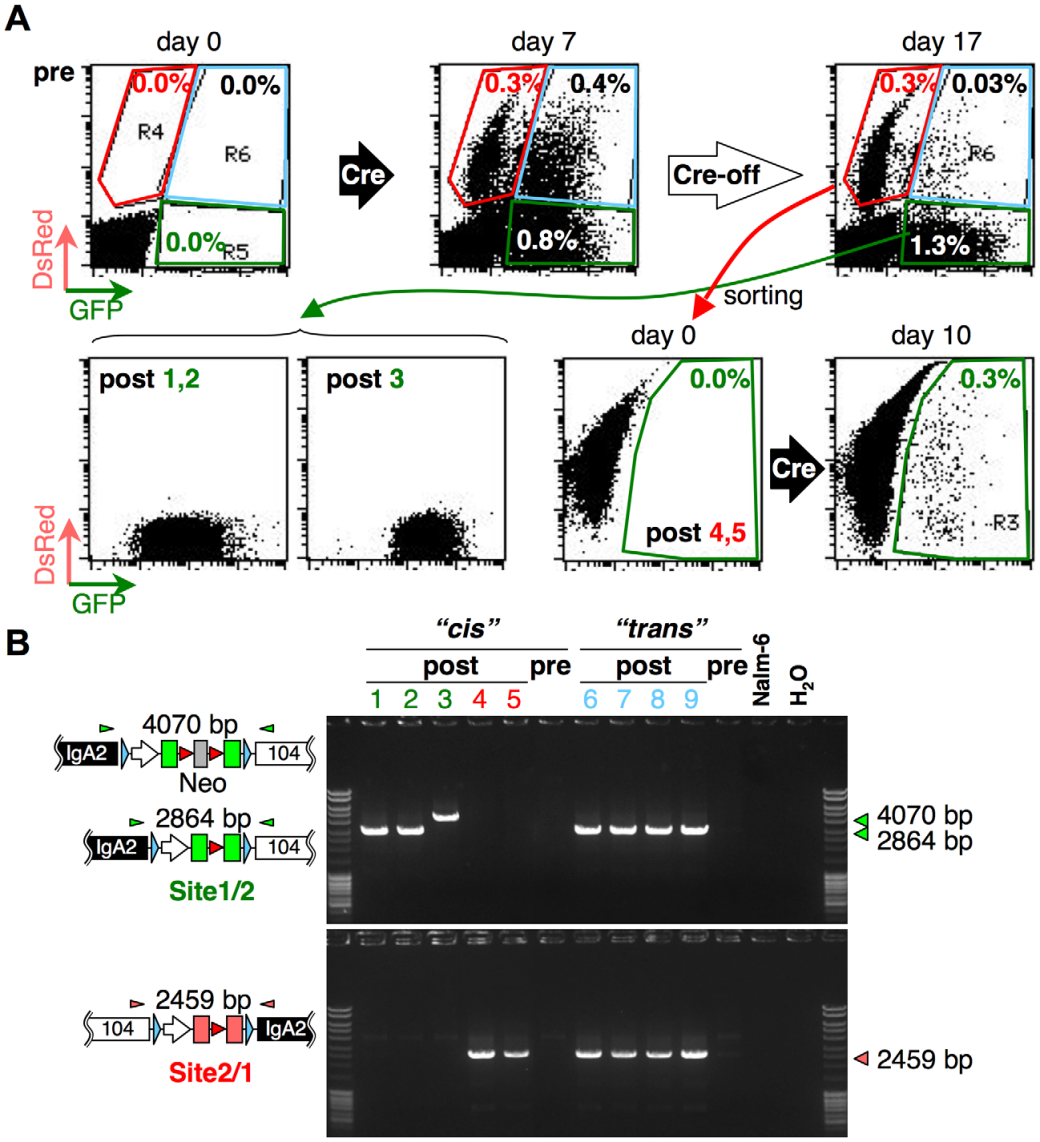
Induction of copy-number variation. A, Flow cytometric profiles of double-targeted cells, Cre-activated cells, and Cre-terminated cells kept OHT-free for 10 days (top). Flow cytometric profiles of sorted cell clones and a DsRed-positive clone 10 days after Cre activation (bottom). B, PCR confirmation of rearrangement for clones from *cis*- and *trans*-targeting cells. Clone numbers from 1 to 5 correspond to rearranged clone numbers (post) in A. Clones 6 to 9 are derived from *trans*-targeted cells. Cells before Cre activation (pre), Nalm-6 and the no-template control (H_2_O) are included. Triangles above the transgene schemes represent primer positions (IgA2-B41-F1 and 104-B41-R1 for green, and 104-B23-F1 and IgA2-B23-R1 for red).

We analyzed double-targeted cells (NSK) selected after Southern blotting (Fig. S5) and metaphase FISH using Site1 and Site2 probes to examine whether Site2 integration occurred in the Site1-integrated chromosome 14 (integration in *cis*) or in its homologue (integration in *trans*) (Fig. 3B, Table 1). The four *cis*-targeted clones were cultured in the presence of OHT. On day 7, cells positive for either GFP or DsRed were observed [(1.5±0.4)×10^−2^; n = 4] (Fig. 4A, top). Cells were passaged in the absence of OHT for an additional 10 days, when the fraction of double-positive cells significantly decreased from 0.4×10^−2^ to 0.3×10^−3^ (Fig. 4A, top; compare day 7 and day 17).

**Table 1:** Targeting configuration of NSK cells with *loxP* sites spanning 1.1 Mb in the same direction.

Targeting configuration	Clone	No. of metaphase	Colocalization	Non-colocalization	Frequency of fluorescence-positive cells
*cis*	NSK122	8	7	1	1.2×10^−2^
	NSK126	7	7	0	1.3×10^−2^
	NSK164	7	7	0	1.6×10^−2^
	NSK171	8	7	1	2.0×10^−2^
	mean frequency (mean ± standard deviation)				1.5±0.4×10^−2^
*trans*	NSK107	7	0	7	3.7×10^−4^
	NSK108	12	1	11	3.0×10^−4^
	NSK114	7	0	7	3.6×10^−4^
	NSK116	16	2	14	1.8×10^−4^
	NSK123	8	1	7	2.5×10^−4^
	NSK145	8	1	7	2.0×10^−4^
	mean frequency of (mean ± standard deviation)				2.8±0.8×10^−4^
mixed	NSK72	7	3	4	3.8×10^−3^
	NSK141	14	6	8	2.3×10^−4^

The numbers of metaphases analyzed and those which exhibited colocalization or non-colocalization of signals by Site1 and Site2 probes are shown with frequencies of double-positive cells after 7 days of OHT stimulation.

To confirm deletion or duplication, we sorted and cloned GFP- and DsRed-positive populations. Among 12 GFP-only clones, 11 clones revealed similar GFP intensity (comparable to post 1, 2 in Fig. 4A bottom), while 1 (post 3) showed higher GFP expression. Profiles of the two DsRed-only clones (post 4, 5) were similar. PCR analysis of genomic DNA of these clones using deletion- or duplication-specific primers produced consistent results, except for the post 3 clone (Fig. 4B), which retained the neomycin-resistant gene that was detected by sequencing the PCR products. Therefore, Cre-mediated excision of drug markers was efficient but incomplete. Culture of DsRed-positive cells in the presence of OHT generated GFP-positive cells, but not vice versa (Fig. 4A, lower right). This observation is consistent with our interpretation that DsRed expression represents cells with inter-*loxP* duplication.

Two clones determined to be mixed (NSK72 and NSK141) by FISH analysis using Site1 and Site2 probes (Table 1) were excluded from the following studies, because these clones may be a mixture of *cis*- and *trans*-targeted clones. However, such a mixed pattern can be explained by cross-hybridization between Site1 and Site2, because identical sequence blocks occupy 29% and 38% of Site1 and Site2 probes, respectively.

### Application to assessment of proximity between gene loci

**Figure 5:**
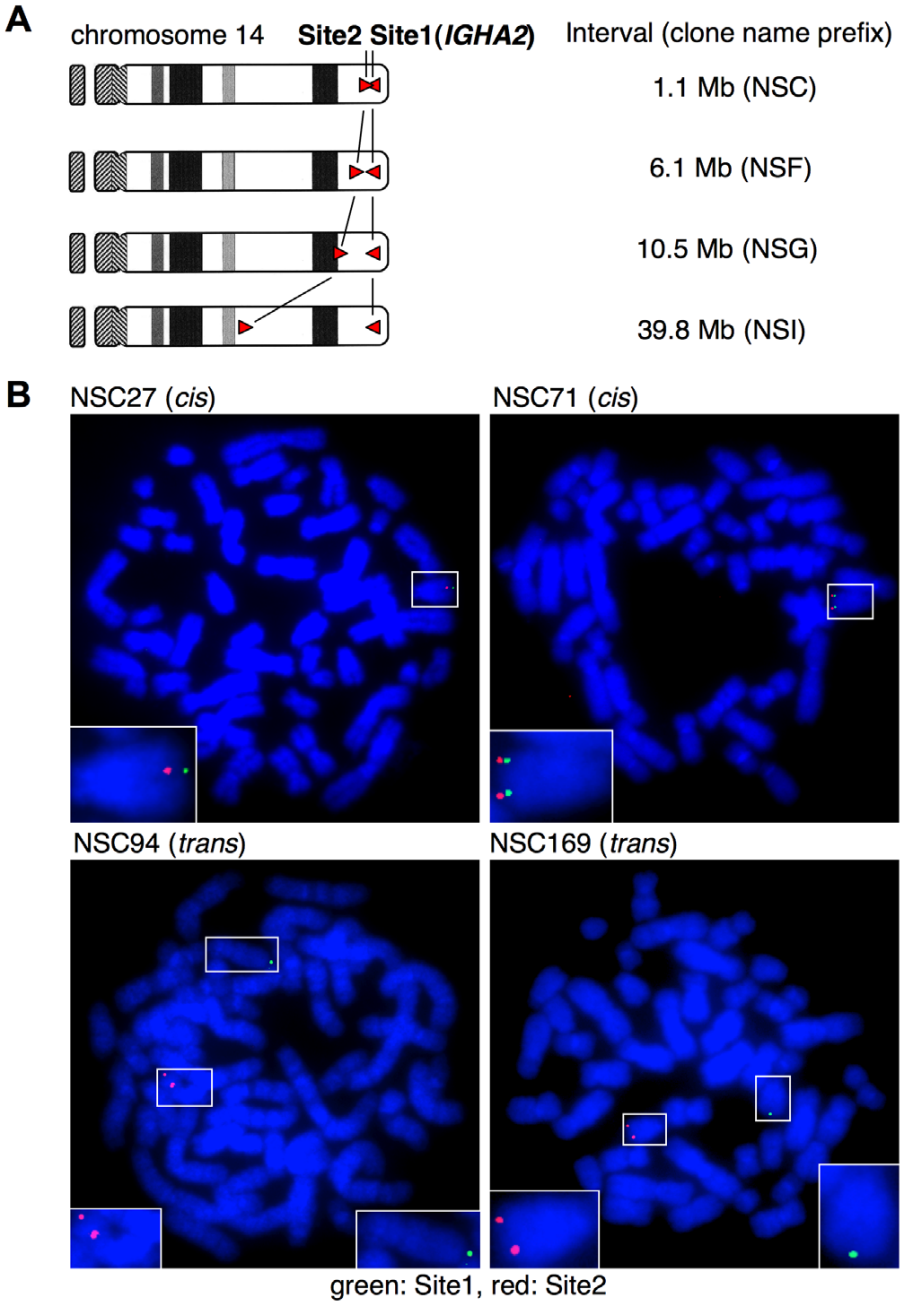
Site1 and Site2 integration for assessment of proximity between loci. A, Scheme of chromosome 14 after *cis* targeting with the indicated Site1–Site2 intervals. B, FISH analyses of representative two *cis*-targeted (NSC27 and NSC71) and two *trans*-targeted clones (NSC94 and NSC169) are shown. The chromosomes 14 with Site1 and Site2 integration harbor green and red signals, respectively, and are highlighted with boxes, magnifications of which are shown in insets.

In somatic cells of fly, homologue pairing (a phenomenon in which paternal and maternal chromosomes spatially align) was demonstrated by Flp-*FRT*-mediated chromosomal recombination [18]. This observation suggests that recombination frequency can be an indicator of proximity between two loci. Similar approach by Cre-*loxP* system has been applied in the yeast chromosome [19]. Frequency of Cre-*loxP*-mediated long-range recombination was shown to decrease with an increase in the inter-*loxP* distance in fly [17] and in mouse embryonic stem cells [6]. To reproduce these results in human cells, we inserted Site2 into Site1-pre-integrated chromosome 14 at intervals of 1.1, 6.1, 10.5 and 39.8 Mb (Fig. 5A). In this experiment, NSB1 cells with Site1 integration at *IGHA2* (106.06 Mb on the chromosome 14 coordinate of the reference human genome build 37.1) were used again. The Site2-targeted loci are located at an intergenic region between *LOC122631* and *C14orf180* (104.94 Mb) for 1.1-Mb interval; an intron of *CCDC85C* (99.99 Mb) for 6.1-Mb interval; *DICER1* (95.57 Mb) for 10.5-Mb interval; and an intergenic region between *FUT8* and *NCOA4P* (66.30 Mb) for 39.8-Mb interval. For inversion to occur, Site2 was oriented in a direction opposite to Site1. This is because cell lethality by deletions of large DNA segments might obscure the Cre recombination frequency [6].

In case of the 1.1-Mb interval (NSC cells), six clones were confirmed to be correctly targeted by Southern blotting (Fig. S6). After 7 days of culture in the presence of OHT, these clones were classified into two groups in terms of frequency of double-positive cells: high group [(9.4±1.1)×10^−3^; *n* = 3] and low group [(8.7±1.0)×10^−5^; *n* = 3]. FISH analysis using Site1 and Site2 probes revealed that high- and low-group clones were *cis*- and *trans*-targeted, respectively (Fig. 5B, Table 2), which also allowed us to classify the high and low groups as *cis* and *trans* groups, respectively, for cells with other Site1–Site2 intervals. In case of the 6.1-Mb interval (Fig. S7), the frequency of double-positive cells after 7 days of OHT stimulation was significantly lower than that seen with the 1.1-Mb interval: (1.2±0.6)×10^−3^ (*cis*, *n* = 4) and (3.0±1.7)×10^−5^ (*trans*, *n* = 8). Likewise, the targeted clones of 10.5- and 39.8-Mb intervals (Fig. S8 and Fig. S9) resulted in further decreasing frequencies: (6.5±3.5)×10^−4^ and (1.6±0.8)×10^−5^ (*cis*, *n* = 4 and *trans*, *n* = 3) for 10.5 Mb and (1.2±0.3)×10^−4^ and (8.9±5.9)×10^−6^ (*cis*, *n* = 4 and *trans*, *n* = 7) for 39.8 Mb.

**Table 2:** Targeting configuration of NSC cells with *loxP* sites spanning 1.1 Mb in the opposite direction.

Targeting configuration	Clone	No. of metaphase	Colocalization	Non-colocalization	Frequency of fluorescence-positive cells
*cis*	NSC27	8	8	0	1.0×10^−2^
	NSC71	8	8	0	8.2×10^−3^
	NSC130	8	7	1	9.6×10^−3^
	mean frequency (mean ± standard deviation)				9.3±0.9×10^−3^
*trans*	NSC94	16	1	15	8.8×10^−5^
	NSC169	8	0	8	7.7×10^−5^
	mean frequency (mean)				8.3×10^−5^

The numbers of metaphases analyzed and those which exhibited colocalization or non-colocalization of signals by Site1 and Site2 probes are shown with frequencies of double-positive cells after 7 days of OHT stimulation.

**Figure 6:**
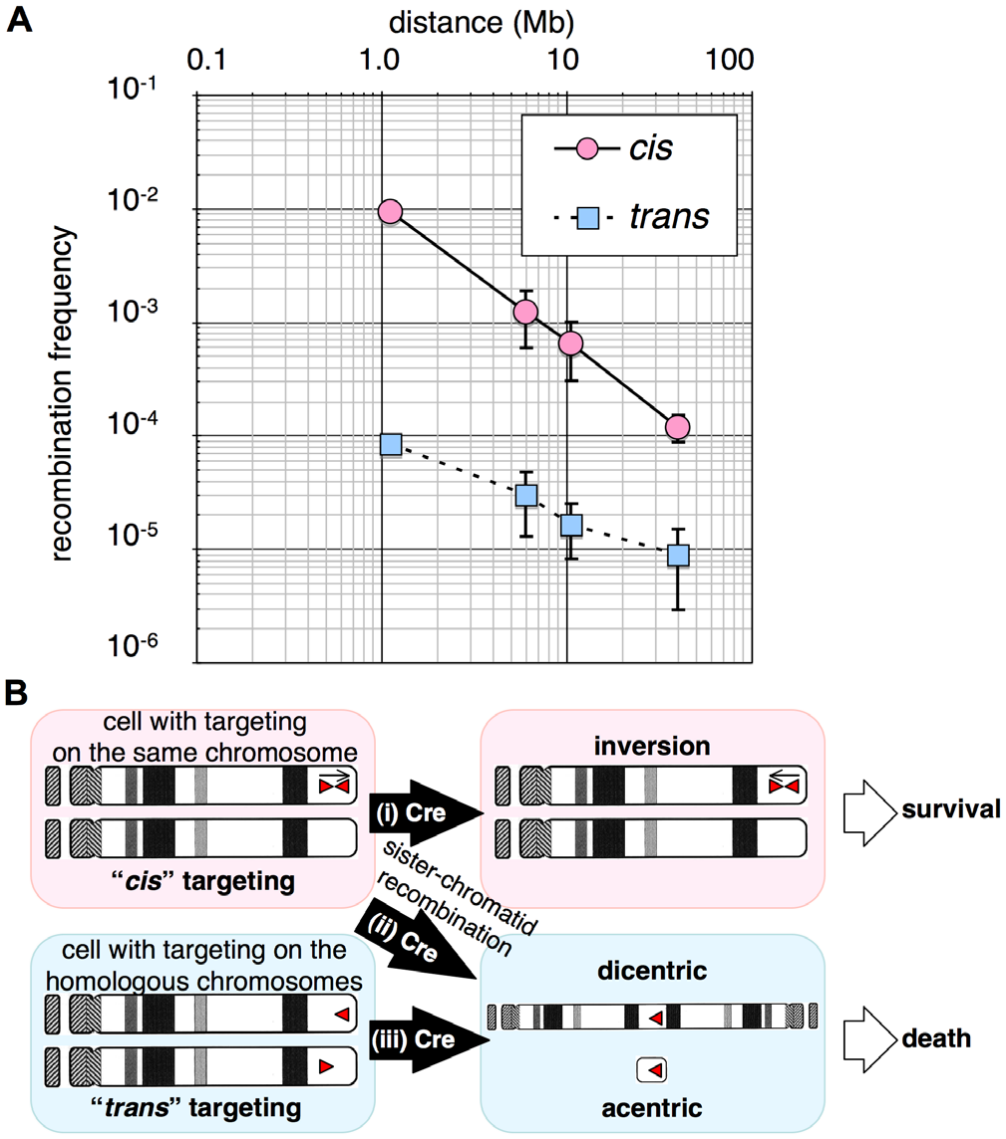
Relationship between recombination frequency and genetic distance. A, Plots of physical distance in the chromosome on the x axis and frequency of recombination on the y axis for *cis*- and *trans*-targeted cells. B, Outcome of inter-*loxP* recombination in *cis*- and *trans*-targeted cells.

**Figure 7:**
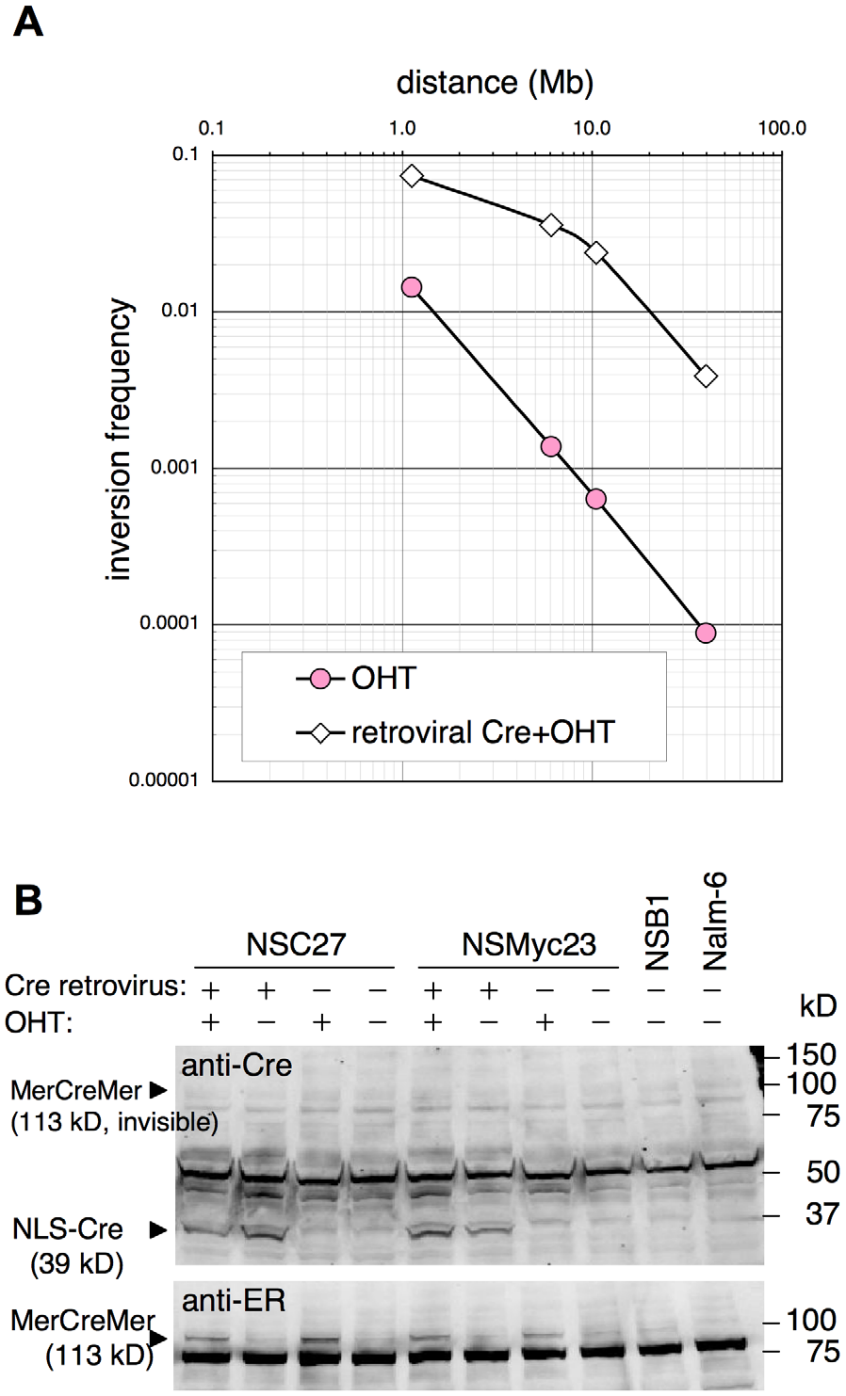
Effect of augmented Cre activity on inversion frequency. A, Representative clones for each *loxP* interval (NSC27 for 1.1 Mb, NSF331 for 6.1 Mb, NSG118 for 10.5 Mb, and NSI95 for 39.8 Mb) were infected with a retrovirus expressing nuclear localization signal (NLS)-tagged Cre and puromycin-resistance gene. Simultaneously, tamoxifen (OHT) was added to activate MerCreMer. One day after infection, 0.25 µg/ml puromycin was added to select infected cells. Retrovirally Cre-transduced cells (open diamond) were analyzed by flow cytometry on day 11 to measure the frequency of fluorescence-positive cells. As a control, non-infected cells (red circle) were stimulated with OHT. B, Western blotting for retrovirally transduced Cre and MerCreMer proteins in NSC27 and NSMyc23 cells, the latter of which is clone #23 used for MYC/IgH translocation induction (Fig. 2B–D and Fig. S2). Retrovirus-infected cells were maintained in the presence of puromycin more than two weeks before lysate preparation.

Plotting the frequencies of recombination versus different *loxP* intervals indicated good linearity (Fig. 6A, 6B-i), suggesting that the intrachromosomal recombination frequency is inversely proportional to the distance between recombination sites. This result is strikingly similar to a result in fly [17] and is consistent with reported observation by Hi-C method and prediction by the fractal globule model of chromatin organization [20]. However, the linearity reduced with an increase in Cre expression provided by a retroviral vector (Fig. 7) probably because multiple inversions underestimate the real recombination frequency higher than 2%. Cre expression in representative clones for each Site1-Site2 interval was augmented by infecting cells with Cre-expressing retrovirus. Retroviral Cre protein but not that of MerCreMer was detected using anti-Cre antibody (Fig. 7, upper panel), while MerCreMer was detected in the presence of OHT by anti-estrogen receptor α antibody. This result indicated that the expression level of retroviral Cre was higher than that of MerCreMer expressed from plasmid transgene.

**Figure 8:**
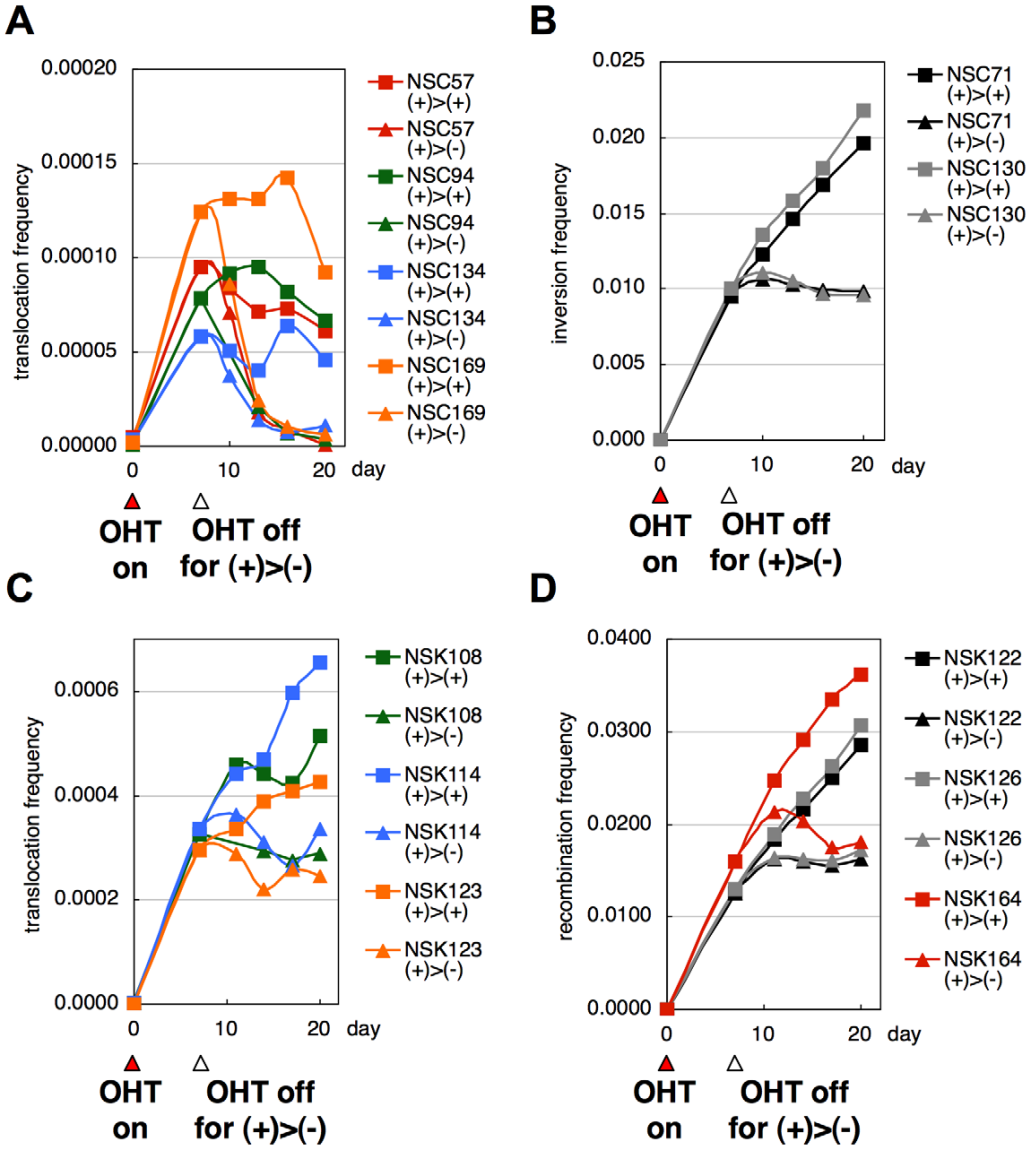
Time course of fluorescence-positive cells in Cre-activated NSC and NSK cells. Seven days after NSC and NSK cells were stimulated with OHT to activate MerCreMer, the culture was split into two: one with continued Cre activation (square) and the other without OHT to terminate Cre activation (triangle). Frequencies of fluorescence-positive cells at the indicated time points were measured by flow cytometry and plotted for NSC (inverted *loxP* sites with a 1.1-Mb interval) *trans*-targeted cells (A) and *cis*-targeted cells (B), and NSK (direct *loxP* sites with a 1.1-Mb interval) *trans*-targeted cells (C) and *cis*-targeted cells (D).

Sister chromatid recombination in *cis*-targeted cells can also lead to cell death associated with dicentric (two-centromere) and acentric (no-centromere) chromosomes (Fig. 6B-ii), which might be another reason for the reduced linearity under high Cre activity. Unexpectedly, the *trans* group also exhibited a decreasing trend according to increasing inter-*loxP* distance (Fig. 6A). In the case of *trans* targeting, recombination results in formation of dicentric and acentric chromosomes, leading to cell death (Fig. 6B-iii). The decreasing trend of the *trans* group could be due to accelerated cell death with longer intervals or homologue pairing as previously observed in fly [18] and yeast [19]. Limited survival of those cells was depicted by the disappearance of the double-positive cells induced by OHT-treatment of *trans*-targeted NSC cells after removal of OHT (Fig. 8A), indicating poor survival of these cells presumably by formation of dicentric and acentric chromosomes (Fig. 6B-iii). In contrast, the double positive cells induced from the *cis* group of NSC cells (Fig. 8B) and those from the NSK cells with *loxP* sites in the same orientation (Fig. 8C for the *trans* group, Fig. 8D for the *cis* group) survived after removal of OHT. This experiment indicates that our system can be applied to studies of spatial organization of the mammalian genome based on an easy and rapid flow-cytometric determination of recombination frequencies.

In this study, we demonstrated the use of our novel system in studying artificial chromosomal translocation, duplication, deletion, and inversion as well as in determining gene proximity in a human cell line. At the same time, this system may be useful for screening of recessive mutations [5] and induction of specific chromosomal loss [21] in cell lines or animal models. The applications to cell lineage tracing and neural connection analysis in animals may also be possible, taking advantage of infrequent recombination between *loxP* sites introduced at identical locations on homologous chromosomes [11]. The technical improvements reported here will facilitate mammalian chromosome engineering and better understanding of human diseases caused by chromosomal translocations and CNV.

## Materials and Methods

### Constructs

Procedures for construction of Site1 and Site2 targeting vectors are described in Methods S1 including Fig. S10, Fig. S11 and Table S1. pANMerCreMer-zeo was constructed by inserting a blunt 1437-bp ClaI-BstPI fragment from pVgRXR (Invitrogen, Carlsbad, CA), containing a zeocin-resistance gene in place of the AatII-SacI neomycin-resistance gene segment of pANMerCreMer-neo [14]. The Flp expression vector pCAGGS-FLPe was purchased (Gene Bridges, Dresden, Germany). A retroviral vector expression Cre, pCre-FBP, was constructed by inserting into pFB vector (Stratagene, La Jolla, CA) a coding sequence of Cre amino-terminally tagged with nuclear localization signal [22] connected with a DNA segment consisting of internal ribosome entry site and puromycin-resistance gene.

### Cell culture, transfection and gene targeting

Nalm-6 cells and derivatives were maintained in ES medium (Nissui, Tokyo, Japan) supplemented with 10% fetal bovine serum, 1× GlutaMAX (Invitrogen) and 50 µM 2-mercaptoethanol. Two micrograms of ScaI-linearized pANMerCreMer-zeo was electroporated into 2×10^6^ Nalm-6 cells using Nucleofector II (Amaxa, Köln, Germany) with Solution T and Program C-005. One of the zeocin-resistant clones with OHT-dependent Cre activity assessed by transient expression of the test substrate was designated as NCR1 to be used in subsequent experiments. The Site1 and Site2 targeting vectors were linearized by one of the enzymes within the multiple linearization sites and introduced into NCR1 and Site1-targeted NCR1, respectively, using Nucleofector II. Drug-resistant colonies were first screened by real-time SYBR-Green PCR with Mx3000P (Stratagene) using genomic DNA isolated by FTA card (Whatman, Kent, UK) as templates. The specific PCR products were detected by a dissociation curve analysis, taking advantage of the fact that the melting temperature of specific PCR products that are ≥2 kb is significantly higher than that of non-specific products including primer dimers. The polymerase was selected from among PrimeSTAR HS, LA Taq HS (Takara, Otsu, Japan), KOD FX (Toyobo, Osaka, Japan) and Phusion (Finnzyme, Espoo, Finland) for the best efficiency and specificity upon amplification using positive control CF and negative control Nalm-6 DNA. Products with melting temperatures similar to CF were almost always had the expected size upon 1% agarose-gel electrophoresis. Positive clones were examined by Southern blotting using upstream, internal and downstream probes labelled with the AlkPhos Direct kit (GE Healthcare, Piscataway, NJ). Detection was performed following 30 min of exposure to LAS-3000 mini cool-CCD camera (Fuji, Tokyo, Japan). Transient expression of pCAGGS-FLPe was obtained using Nucleofector II.

### Fluorescence *in situ* hybridization

We performed two-color FISH analysis using two BAC clones RP11-953L20 (located centromeric to the *IgH* gene; SpectrumGreen-labeled) and RP11-55J15 (located telomeric to the *MYC* gene; SpectrumOrange-labeled). Labeling reaction was done using Nick Translation Kit (Abbott Molecular Inc., Des Plaines, IL) and SpectrumGreen- and SpectrumOrange-labeled deoxyuridine triphosphate (Abbott Molecular Inc.). For discrimination of *cis* and *trans* targeting, we used PCR-amplified Site1 (3.8 kb) and Site2 (2.9 kb) probes labelled with SpectrumGreen and SpectrumOrange, respectively. Images were taken with the fluorescence microscope MD5000B (Leica, Wetzlar, Germany), equipped with a cool charge-coupled device camera ORCA-ER (Hamamatsu Photonics, Hamamatsu, Japan) and iVision-Mac software (BioVision Technologies, Exton, PA). For spectral karyotyping of *BCR/ABL1* translocation, hybridization was done using a SKYPaint kit (Applied Spectral Imaging, Vista, CA) and detected by SD-200 (Applied Spectral Imaging).

### Induction of recombination and flow cytometry

After double targeting, 100,000 Nalm-6 cells were inoculated in 1 ml of medium with or without 1 µM OHT (day 0). On day 3, 0.5 ml of cells was diluted to 5 ml while maintaining OHT concentrations. Flow-cytometric analyses were performed by FACSCalibur (Becton Dickinson, Franklin Lakes, NJ) on day 7 in most cases. In some cases, this was done on day 11 or later, in which case the cells were diluted 10 times every 3 to 4 days. The frequencies of fluorescence-positive cells were then calculated from data acquired for one million cells using CellQuest software (Becton Dickinson). When OHT-stimulated cells were to be sorted, culture volume was doubled. Sorting was performed using FACSCalibur.

### Western blotting

We performed western blot analysis for the BCR-ABL1 fusion protein using mouse monoclonal antibody against c-Abl [c-Abl(24-11); Santa Cruz, Santa Cruz, CA] or GAPDH (6C1, Millipore, Billerica, MA) and IR Dye800CW-labelled anti-mouse IgG (Li-cor, Lincoln, NE) and detected the proteins using a Odyssey scanner (Li-cor). Cre was detected using mouse monoclonal antibody (7.23; Abcam, Cambridge, MA) and IRDye680CW-labelled anti-mouse IgG (Li-cor). MerCreMer was detected with rabbit polyclonal anti-human estrogen receptor α antibody (sc-543, Santa Cruz) and IR Dye800CW-labelled anti-rabbit IgG (Li-cor). Antibody incubation was 10 min at room temperature using the SNAPi.d. Protein Detection System (Millipore).

## Supporting Information

Methods S1 Construction of Site1 and Site2 targeting vector.
(0.03 MB RTF)

Table S1 Oligonucleotide sequences. “P-” indicates 5′ phosphorylation. PCR enzymes used to amplifiy DNA fragments are shown. Blue letters indicate relevant ristriction sites. Green letters indicate SV40 small-t intronic sequence. Red letters indicate *att* site sequences.
(0.04 MB XLS)

Figure S1 Gene targeting strategy for *IGHA2* locus and screening summary. A, Structure of the *IGHA2* loci encoding the constant region of IgA2 before and after targeting (upper panel) and expected band sizes on the Southern blot (table). Rectangles indicate exons and the oval shape indicates the switch region of the *IGHA2* locus (Sα 2). Exon numbers are indicated in white. Regions of targeting homology are indicated by parallel dotted lines with in-between numbers depicting bp. Positions of DNA probes used for Southern blotting are indicated by thick blue (upstream), black (internal) and red (downstream) lines. Numbers with bp above bidirectional arrows represent the distance between relevant restriction sites. Primer positions for PCR screening are indicated by arrowheads. Unidirectional arrows indicate direction toward the centromere or the telomere. The table lists the expected sizes of bands on the Southern blotting using the indicated enzymes and probes. Due to high homology between the *IGHA2* and the *IGHA1* loci encoding IgA1, expected signals from both loci are shown for wild-type (WT) and targeted (knockout, KO) alleles. B, Southern blot using the indicated enzymes and probes for representative five clones (#138, #148, #159, #201 and #209). Genomic DNA of these clones and their subclones (1–3) with parental NCR1 cells were analyzed. Outermost lanes of each panel were loaded with a size marker. The expected positions of WT and KO alleles are shown by arrowheads. The PvuII fragments detected by the upstream probe from the *IGHA1* locus showed allelic polymorphism, and are tentatively designated a and b alleles. The longer a allele appeared to lose an upstream PvuII site in the allele b fragment. C, Summary of clone numbers is indicated. Subclone 1 of clone #159 was designated NSB1, which was subsequently targeted in all experiments except induction of *BCR/ABL1* translocation.
(1.83 MB TIF)

Figure S2 Gene targeting strategy for *MYC* locus and screening summary. A, Structure of the *MYC* loci before and after targeting (upper panel) and expected band sizes on the Southern blot (table). Rectangles indicate exons of the *MYC* gene. Exon numbers are indicated in white. Regions of targeting homology are indicated by parallel dotted lines with in-between numbers depicting bp. Positions of DNA probes used for Southern blotting are indicated by thick blue (upstream), black (internal) and red (downstream) lines. Numbers with bp above bidirectional arrows represent the distance between relevant restriction sites. Primer positions for PCR screening are indicated by arrowheads. Unidirectional arrows indicate direction toward the centromere or the telomere. The table lists the expected sizes of bands on the Southern blot obtained using the indicated enzymes and probes for wild-type (WT) and targeted (knockout, KO) alleles. B, Southern blot using the indicated enzymes and probes for eight clones and parental NSB1 cells. Outermost lanes of each panel were loaded with the size marker. Expected positions of WT and KO alleles are shown by arrowheads. C, Summary of clone numbers is indicated.
(2.78 MB TIF)

Figure S3 Gene targeting strategy for *BCR* locus and screening summary. A, Structure of the *BCR* loci before and after targeting (upper panel) and expected band sizes on the Southern blot (table). Rectangles indicate exons of the *BCR* gene. Exon numbers are indicated in white. Regions of targeting homology are indicated by parallel dotted lines with in-between numbers depicting bp. Positions of DNA probes used for Southern blotting are indicated by thick blue (upstream), black (internal) and red (downstream) lines. Numbers with bp above bidirectional arrows represent the distance between relevant restriction sites. Primer positions for PCR screening are indicated by arrowheads. Unidirectional arrows indicate direction toward the centromere or the telomere. The table lists the expected sizes of bands on the Southern blot using the indicated enzymes and probes for wild-type (WT) and targeted (knockout, KO) alleles. B, Southern blot using the indicated enzymes and probes for 14 clones and parental NCR1 cells. Outermost lanes of each panel were loaded with the size marker. Expected positions of WT and KO alleles are shown by arrowheads. Clone #177 (NSE177) was subsequently targeted with an *ABL1*-targeting vector. C, Summary of clone numbers is indicated.
(1.20 MB TIF)

Figure S4 Gene targeting strategy for *ABL1* locus and screening summary. A, Structure of the *ABL1* loci before and after targeting (upper panel) and expected band sizes on the Southern blot (table). Rectangles indicate exons of the *ABL1* gene. Exon numbers are indicated in white. Regions of targeting homology are indicated by parallel dotted lines with in-between numbers depicting bp. Positions of DNA probes used for Southern blotting are indicated by thick blue (upstream), black (internal) and red (downstream) lines. Numbers with bp above bidirectional arrows represent the distance between relevant restriction sites. Primer positions for PCR screening are indicated by arrowheads. Unidirectional arrows indicate direction toward the centromere or the telomere. The table lists the expected sizes of bands on the Southern blot using the indicated enzymes and probes for wild-type (WT) and targeted (knockout, KO) alleles. B, Southern blot using the indicated enzymes and probes for 10 clones and parental NSE177 cells. Outermost lanes of each panel were loaded with the size marker. Expected positions of WT and KO alleles are shown by arrowheads. C, Summary of clone numbers is indicated.
(2.81 MB TIF)

Figure S5 Gene targeting strategy for *104* locus for deletion/duplication and screening summary. A, EcoRI restriction map of a region at 104 Mb on the coordinate of chromosome 14, 1.1 Mb centromeric to the *IGHA2* loci (upper panel), and expected band sizes on the Southern blot (table). Regions of targeting homology are indicated by parallel dotted lines with in-between numbers depicting bp. Positions of DNA probes used for Southern blotting are indicated by thick blue (upstream), black (internal) and red (downstream) lines. Numbers with bp above bidirectional arrows represent the distance between relevant restriction sites. Primer positions for PCR screening are indicated by arrowheads. Unidirectional arrows indicate direction toward the centromere or the telomere. The table lists expected sizes of bands on the Southern blot using the indicated enzymes and probes for wild-type (WT) and targeted (knockout, KO) alleles. B, Southern blot using the indicated enzymes and probes for 14 (NSK) clones and parental NSB1 cells. Outermost lanes of each panel were loaded with the size marker. Expected positions of WT and KO alleles are shown by arrowheads. Two extra bands on the upstream probe blot indicated by open arrowheads and asterisks appeared to be derived from a highly homologous sequence on chromosome 13. C, Summary of clone numbers is indicated.
(1.94 MB TIF)

Figure S6 Gene targeting strategy for *104* locus for inversion and screening summary. A, EcoRI restriction map of a region at 104 Mb on the coordinate of chromosome 14, 1.1 Mb centromeric to the *IGHA2* loci (upper panel), and expected band sizes on the Southern blot (table). Regions of targeting homology are indicated by parallel dotted lines with in-between numbers depicting bp. Positions of DNA probes used for Southern blotting are indicated by thick blue (upstream) and red (downstream) lines. Numbers with bp above bidirectional arrows represent the distance between relevant restriction sites. Primer positions for PCR screening are indicated by arrowheads. Unidirectional arrows indicate direction toward the centromere or the telomere. The table lists the expected sizes of bands on the Southern blotting using the indicated enzymes and probes for wild-type (WT) and targeted (knockout, KO) alleles. B, Southern blot using the indicated enzymes and probes for 11 (NSC) clones and parental NSB1 cells. Outermost lanes of each panel were loaded with the size marker. Expected positions of WT and KO alleles are shown by arrowheads. C, Summary of clone numbers is indicated.
(1.86 MB TIF)

Figure S7 Gene targeting strategy for *99* locus for inversion and screening summary. A, EcoRI restriction map of a region at 99 Mb on the coordinate of chromosome 14, 6.1 Mb centromeric to the *IGHA2* loci (upper panel), and expected band sizes on the Southern blotting (table). Regions of targeting homology are indicated by parallel dotted lines with in-between numbers depicting bp. Positions of DNA probes used for Southern blotting are indicated by thick blue (upstream), black (internal) and red (downstream) lines. Numbers with bp above bidirectional arrows represent the distance between relevant restriction sites. Primer positions for PCR screening are indicated by arrowheads. Unidirectional arrows indicate direction toward the centromere or the telomere. The table lists the expected sizes of bands on the Southern blot using the indicated enzymes and probes for wild-type (WT) and targeted (knockout, KO) alleles. B, Southern blot using the indicated enzymes and probes for 13 (NSF) clones and parental NSB1 cells. Outermost lanes of each panel were loaded with the size marker. Expected positions of WT and KO alleles are shown by arrowheads. C, Summary of clone numbers is indicated.
(1.95 MB TIF)

Figure S8 Gene targeting strategy for *95* locus for inversion and screening summary. A, Restriction map of the *DICER1* locus at 95 Mb on the coordinate of chromosome 14, 10.5 Mb centromeric to the *IGHA2* loci (upper panel), and expected band sizes on the Southern blot (table). Rectangles indicate exons of the *DICER1* gene. Some of exon numbers are indicated in white. Regions of targeting homology are indicated by parallel dotted lines with in-between numbers depicting bp. Positions of DNA probes used for Southern blotting are indicated by thick blue (upstream), black (internal) and red (downstream) lines. Numbers with bp above bidirectional arrows represent the distance between relevant restriction sites. Primer positions for PCR screening are indicated by arrowheads. Unidirectional arrows indicate direction toward the centromere or the telomere. The table lists expected sizes of bands on the Southern blot using the indicated enzymes and probes for wild-type (WT) and targeted (knockout, KO) alleles. B, Southern blot using the indicated enzymes and probes for 16 (NSG) clones and parental NSB1 cells. Outermost lanes of each panel were loaded with the size marker. Expected positions of WT and KO alleles are shown by arrowheads. C, Summary of clone numbers is indicated.
(2.94 MB TIF)

Figure S9 Gene targeting strategy for *65* locus for inversion and screening summary. A, Restriction map of a region at 65 Mb on the coordinate of chromosome 14, 39.8 Mb centromeric to the *IGHA2* loci (upper panel), and expected band sizes on the Southern blot (table). Regions of targeting homology are indicated by parallel dotted lines with in-between numbers depicting bp. Positions of DNA probes used for Southern blotting are indicated by thick blue (upstream), black (internal) and red (downstream) lines. Numbers with bp above bidirectional arrows represent the distance between relevant restriction sites. Primer positions for PCR screening are indicated by arrowheads. Unidirectional arrows indicate direction toward the centromere or the telomere. The table lists the expected sizes of bands on the Southern blot using the indicated enzymes and probes for wild-type (WT) and targeted (knockout, KO) alleles. B, Southern blot using the indicated enzymes and probes for 13 (NSI) clones and parental NSB1 cells. Outermost lanes of each panel were loaded with the size marker. Expected positions of WT and KO alleles are shown by arrowheads. C, Summary of clone numbers is indicated.
(3.14 MB TIF)

Figure S10 Construction scheme part 1. For details, see Methods S1.
(0.61 MB TIF)

Figure S11 Construction scheme part 2. For details, see Methods S1.
(1.74 MB TIF)
