# Supplementary material for: Chromosomal Manipulation by Site-Specific Recombinases and Fluorescent Protein-Based Vectors
Source: PLoS One. 2010 Mar 24;5(3):e9846. doi: 10.1371/journal.pone.0009846 (PMC2844420; doi:10.1371/journal.pone.0009846)
Supplement: Methods S1 — Construction of Site1 and Site2 targeting vector. (0.03 MB RTF) [file pone.0009846.s001.rtf]

Methods S1
Construction of Site1 and Site2 targeting vector
The intron-containing EGFP expression vector pEGFP-intron was created by modifying of pEGFP-N1 (Clontech, Mountain View, CA). SV40 small t gene-derived intronic sequence (71 bp) was included as the 5' extension of PCR primers. Using pEGFP-N1 as a template, PCR was performed using the primers EGFP-Int-1F and EGFP-Int-1R and Phusion enzyme (Finnzyme, Espoo, Finland) to obtain first half of GFP fragment with an intronic sequence starting from the splice donor and ending at the BssHII site. Second half of the GFP fragment preceded by BssHII site and the rest of the intron was similarly obtained using EGFP-Int-2F and EGFP-Int-2R. Sequences of primers are listed in Table S1. The two fragments thus obtained were inserted between BamHI and NotI sites of pEGFP-N1, replacing the original EGFP sequence with that of intron-containing EGFP. Likewise, the intron-containing DsRed expression vector pDimer2-intron was created using pEB6CAGdimer2 (a gift from Yoshihiro Miwa that contained the dimer2 coding sequence originally developed by Roger Tsien) as a template and pEGFP-N1 as a plasmid backbone, using two pairs of primers—DsRed-Int-3F and DsRed-Int-3R, and DsRed-Int-4F and DsRed-Int-4R. BssHII-NotI fragments from pEGFP-intron and pDimer2-intron were swapped to generate pEGFmer2b and pDiPb (Fig. S10). After removal of four restriction sites by blunt-end ligation and conversion of the BssHII site to the XhoI site by insertion of an annealed BXB linker, pEGFmer2x2 and pDiPx2 were obtained. A floxed neomycin-resistance marker was excised as a SalI fragment from the plasmid pNeo-Flox1. This plasmid was prepared from pMC1neoPolyA (Stratagene, La Jolla, CA) and pFlox1.4, which is a modified pBluescript (Stratagene) with two tandem loxP sites (K.K., unpublished). The floxed hygromycin-resistance plasmid pHyg-Flox1 was prepared from pPGKhygro and pFlox1.4. The SalI fragments of floxed drug-resistance markers were inserted into pEGFmer2x2 and pDiPx2 to generate pEGFmer2-H and pDiP-N, respectively. Adding the XhoI site beside the PshBI site by insertion of the PXP linker and the SalI site beside the AflII site by insertion of the ASA linker for pEGFmer2-H and pDiP-N, pSite3 and pSite4 were created, respectively (Fig. S10).
The AatII-AflIII fragment (1815 bp) from pBR322 was ligated with two fragments of annealed 5'-phosphorylated oligonucleotides (FRT1P and FRT2P) to generate pFrt1 with two tandem FRT sites and three restriction sites consisting of central EcoRI and flanking XhoI and SalI sites (Fig. S11). Utilizing XhoI and SalI sites, annealed attB1 and attB2 oligos were sequentially inserted to generate pFrt3. XhoI-SalI fragments from pSite3 and pSite4 were inserted into the EcoRI site of pFrt3 to generate pSite1 and pSite2. The reverse orientation products pSite5 and pSite6 were also obtained. Recombination between these plasmids and pDONR221 of the Gateway system (Invitrogen, Carlsbad, CA) by BP Clonase produced Gateway entry vectors, pENTR-Site1, pENTR-Site2, pENTR-Site5 and pENTR-Site6 (Fig. S11).
In this study, 8 loci including chromosome 14 regions of 105 Mb (IGHA2), 104 Mb, 99 Mb, 95 Mb (DICER1), and 65 Mb on the chromosomal coordinate; the MYC locus on chromosome 8; the BCR locus on chromosome 22, and the ABL1 locus on chromosome 9 were targeted. Since the targeting arms for the 104-Mb region on chromosome 14 were combined with Site2 and Site6 (inverted Site2) vectors, 9 targeting vectors were prepared. Sequences of the primers used for preparation of 5'- and 3'-arm vectors are listed in Table S1. Targeting arms with attB sites were PCR amplified using specific primers 5'-extended with attB sequences, genomic DNA of Nalm-6 cells and PrimerSTAR HS polymerase (Takara, Otsu, Japan) after 35 cycles. Gel-purified PCR products were cloned into pDONR P4-P1R (Invitrogen) or pDONR P2R-P3 (Invitrogen) by BP Clonase (Invitrogen) to obtain two Gateway entry clones of 5'- and 3'-arm vectors. Three entry plasmids (a Site vector plus two arm vectors) and one destination vector (pDEST DTA-MLS, reference 12 of the main text) were assembled by LR Clonase (Invitrogen) to obtain final gene targeting vectors according to manufacturer's instructions. Sequences of targeting arms and cloning junctions were confirmed by sequencing on an ABI3130xl sequencer (Applied Biosystems, Foster City, CA) with a Bigdye Terminator v1.1 reagent set (Applied Biosystems). Positive controls for PCR screening (control fragment, CF) were prepared by PCR using Nalm-6 genomic DNA, a primer located outside the homology region (out) and another primer (long) composed of an internal sequence within the Site core region as well as a sequence from the same homology arm. Actual screening used only the out primer and the third primer of the same sequence within the Site core region (internal). Probes used for Southern blotting were prepared by PCR using Nalm-6 genomic DNA and specific primers outside the homology regions as indicated in Table S1. Plasmids and nucleotide sequences of Site1 and Site2 vectors are available from Addgene (http://www.addgene.org/).
