# Supplementary figures and images for: Chromosomal Manipulation by Site-Specific Recombinases and Fluorescent Protein-Based Vectors
Source: PLoS One. 2010 Mar 24;5(3):e9846. doi: 10.1371/journal.pone.0009846 (PMC2844420; doi:10.1371/journal.pone.0009846)

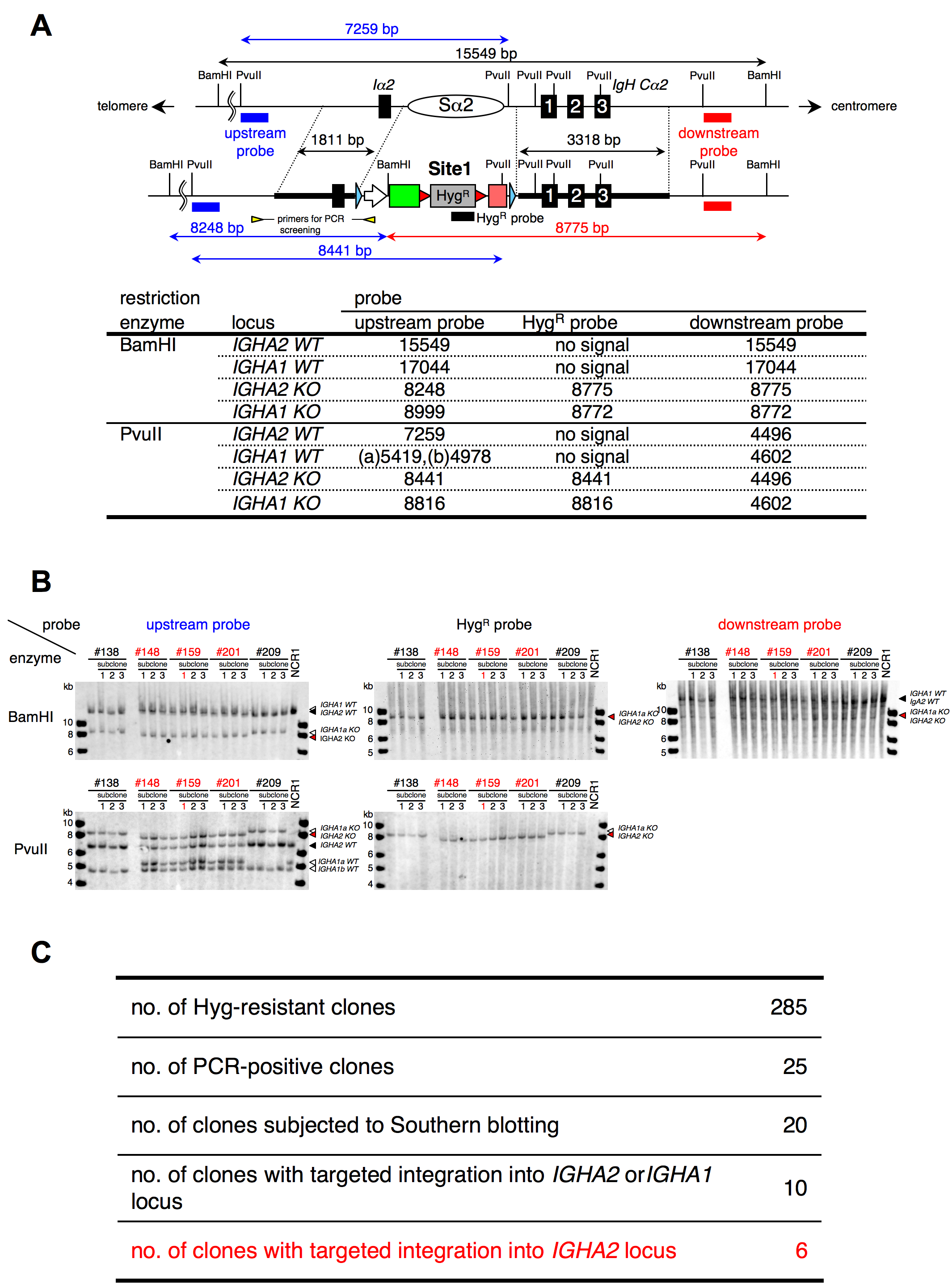

Supplement: Figure S1 — Gene targeting strategy for IGHA2 locus and screening summary. A, Structure of the IGHA2 loci encoding the constant region of IgA2 before and after targeting (upper panel) and expected band sizes on the Southern blot (table). Rectangles indicate exons and the oval shape indicates the switch region of the IGHA2 locus (Sα 2). Exon numbers are indicated in white. Regions of targeting homology are indicated by parallel dotted lines with in-between numbers depicting bp. Positions of DNA probes used for Southern blotting are indicated by thick blue (upstream), black (internal) and red (downstream) lines. Numbers with bp above bidirectional arrows represent the distance between relevant restriction sites. Primer positions for PCR screening are indicated by arrowheads. Unidirectional arrows indicate direction toward the centromere or the telomere. The table lists the expected sizes of bands on the Southern blotting using the indicated enzymes and probes. Due to high homology between the IGHA2 and the IGHA1 loci encoding IgA1, expected signals from both loci are shown for wild-type (WT) and targeted (knockout, KO) alleles. B, Southern blot using the indicated enzymes and probes for representative five clones (#138, #148, #159, #201 and #209). Genomic DNA of these clones and their subclones (1–3) with parental NCR1 cells were analyzed. Outermost lanes of each panel were loaded with a size marker. The expected positions of WT and KO alleles are shown by arrowheads. The PvuII fragments detected by the upstream probe from the IGHA1 locus showed allelic polymorphism, and are tentatively designated a and b alleles. The longer a allele appeared to lose an upstream PvuII site in the allele b fragment. C, Summary of clone numbers is indicated. Subclone 1 of clone #159 was designated NSB1, which was subsequently targeted in all experiments except induction of BCR/ABL1 translocation. (1.83 MB TIF) [file pone.0009846.s003.tif]

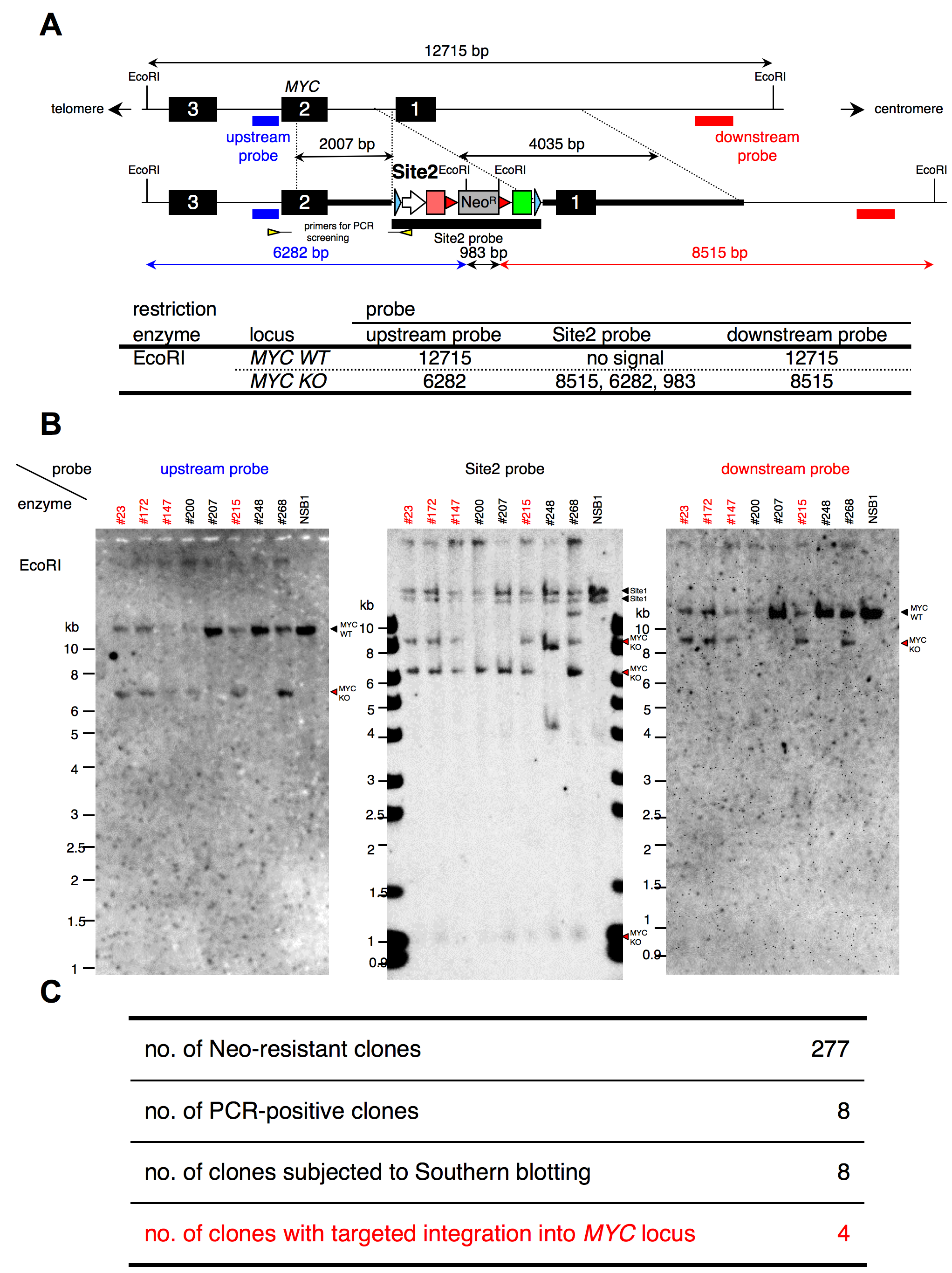

Supplement: Figure S2 — Gene targeting strategy for MYC locus and screening summary. A, Structure of the MYC loci before and after targeting (upper panel) and expected band sizes on the Southern blot (table). Rectangles indicate exons of the MYC gene. Exon numbers are indicated in white. Regions of targeting homology are indicated by parallel dotted lines with in-between numbers depicting bp. Positions of DNA probes used for Southern blotting are indicated by thick blue (upstream), black (internal) and red (downstream) lines. Numbers with bp above bidirectional arrows represent the distance between relevant restriction sites. Primer positions for PCR screening are indicated by arrowheads. Unidirectional arrows indicate direction toward the centromere or the telomere. The table lists the expected sizes of bands on the Southern blot obtained using the indicated enzymes and probes for wild-type (WT) and targeted (knockout, KO) alleles. B, Southern blot using the indicated enzymes and probes for eight clones and parental NSB1 cells. Outermost lanes of each panel were loaded with the size marker. Expected positions of WT and KO alleles are shown by arrowheads. C, Summary of clone numbers is indicated. (2.78 MB TIF) [file pone.0009846.s004.tif]

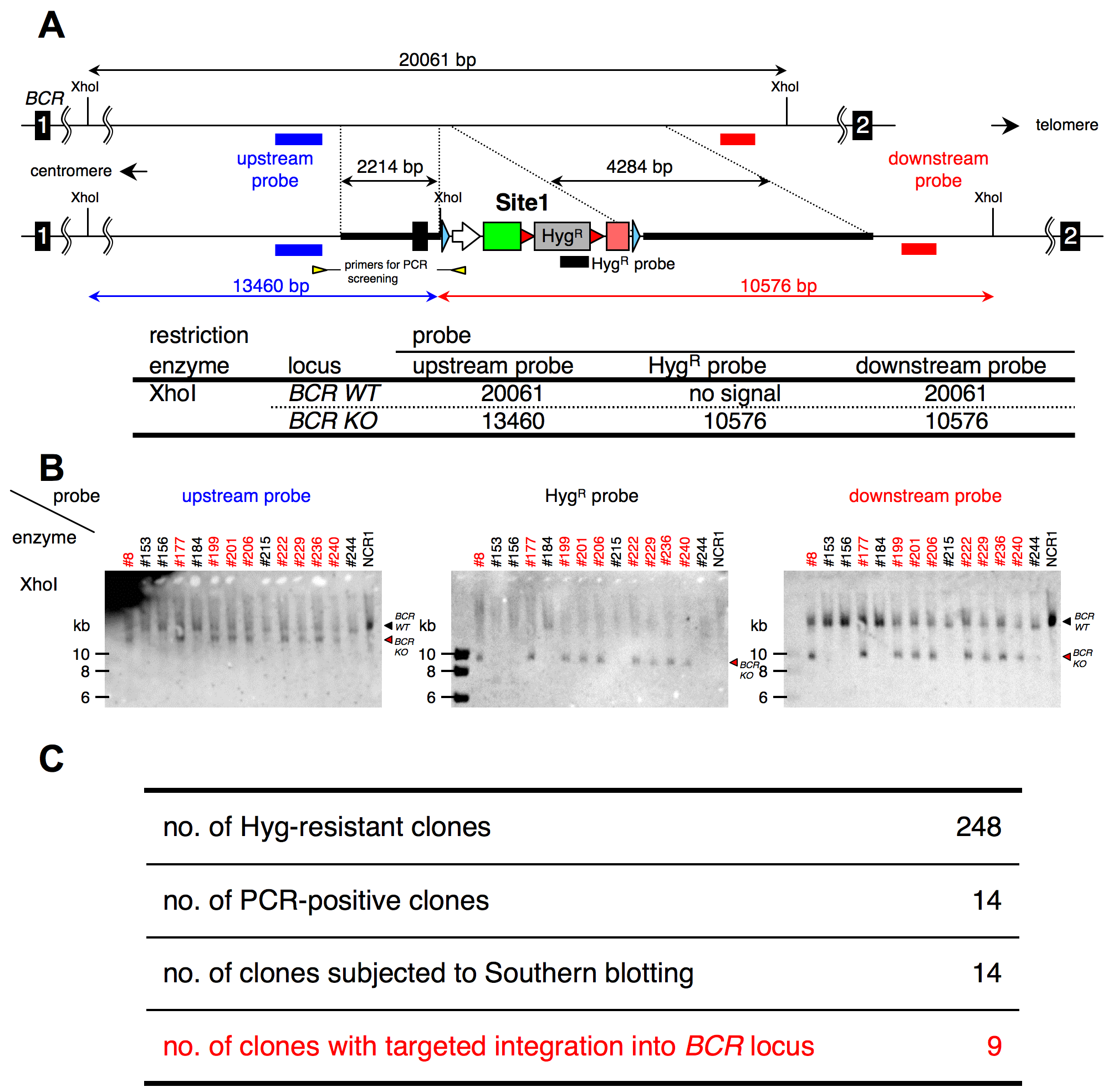

Supplement: Figure S3 — Gene targeting strategy for BCR locus and screening summary. A, Structure of the BCR loci before and after targeting (upper panel) and expected band sizes on the Southern blot (table). Rectangles indicate exons of the BCR gene. Exon numbers are indicated in white. Regions of targeting homology are indicated by parallel dotted lines with in-between numbers depicting bp. Positions of DNA probes used for Southern blotting are indicated by thick blue (upstream), black (internal) and red (downstream) lines. Numbers with bp above bidirectional arrows represent the distance between relevant restriction sites. Primer positions for PCR screening are indicated by arrowheads. Unidirectional arrows indicate direction toward the centromere or the telomere. The table lists the expected sizes of bands on the Southern blot using the indicated enzymes and probes for wild-type (WT) and targeted (knockout, KO) alleles. B, Southern blot using the indicated enzymes and probes for 14 clones and parental NCR1 cells. Outermost lanes of each panel were loaded with the size marker. Expected positions of WT and KO alleles are shown by arrowheads. Clone #177 (NSE177) was subsequently targeted with an ABL1-targeting vector. C, Summary of clone numbers is indicated. (1.20 MB TIF) [file pone.0009846.s005.tif]

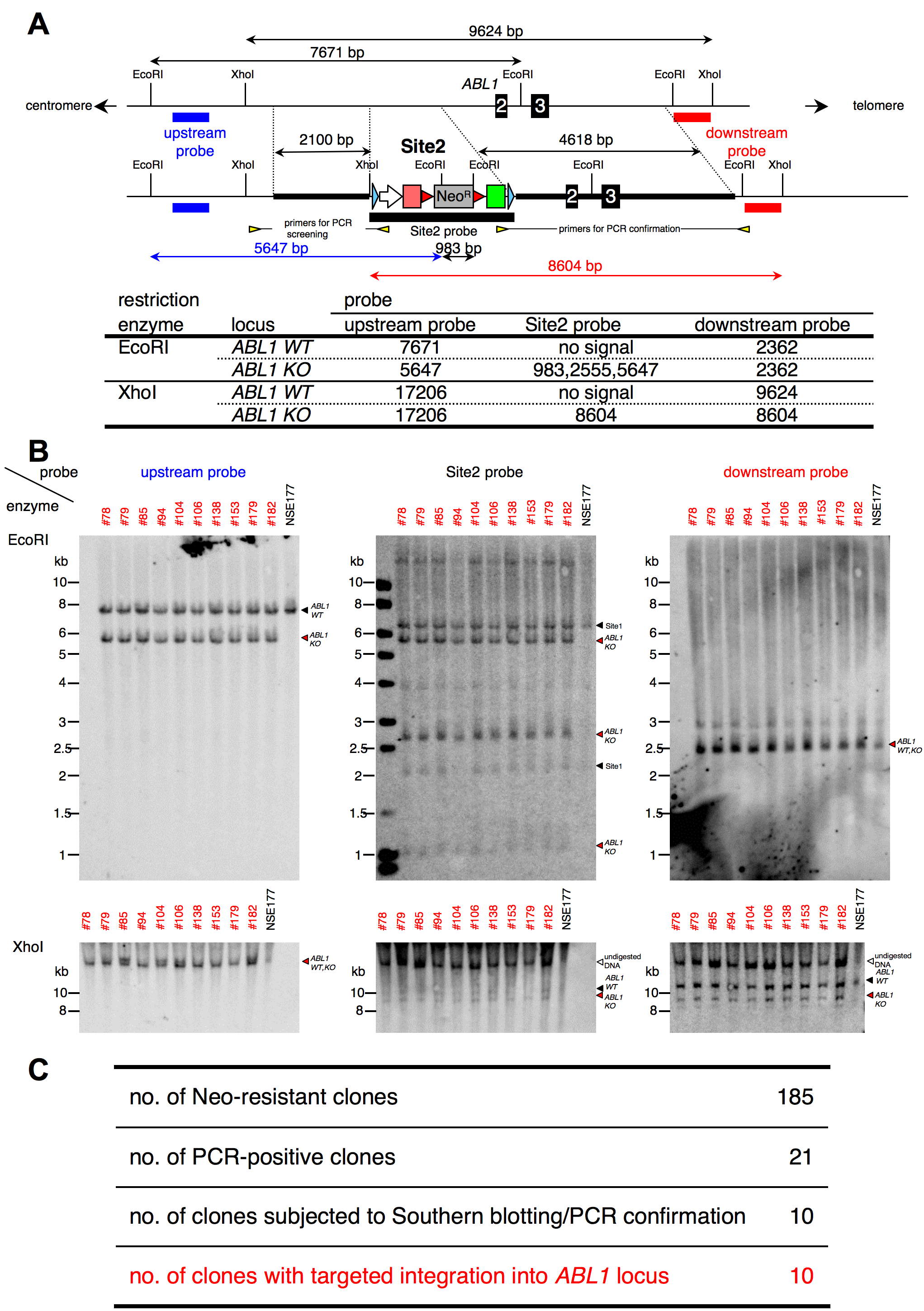

Supplement: Figure S4 — Gene targeting strategy for ABL1 locus and screening summary. A, Structure of the ABL1 loci before and after targeting (upper panel) and expected band sizes on the Southern blot (table). Rectangles indicate exons of the ABL1 gene. Exon numbers are indicated in white. Regions of targeting homology are indicated by parallel dotted lines with in-between numbers depicting bp. Positions of DNA probes used for Southern blotting are indicated by thick blue (upstream), black (internal) and red (downstream) lines. Numbers with bp above bidirectional arrows represent the distance between relevant restriction sites. Primer positions for PCR screening are indicated by arrowheads. Unidirectional arrows indicate direction toward the centromere or the telomere. The table lists the expected sizes of bands on the Southern blot using the indicated enzymes and probes for wild-type (WT) and targeted (knockout, KO) alleles. B, Southern blot using the indicated enzymes and probes for 10 clones and parental NSE177 cells. Outermost lanes of each panel were loaded with the size marker. Expected positions of WT and KO alleles are shown by arrowheads. C, Summary of clone numbers is indicated. (2.81 MB TIF) [file pone.0009846.s006.tif]

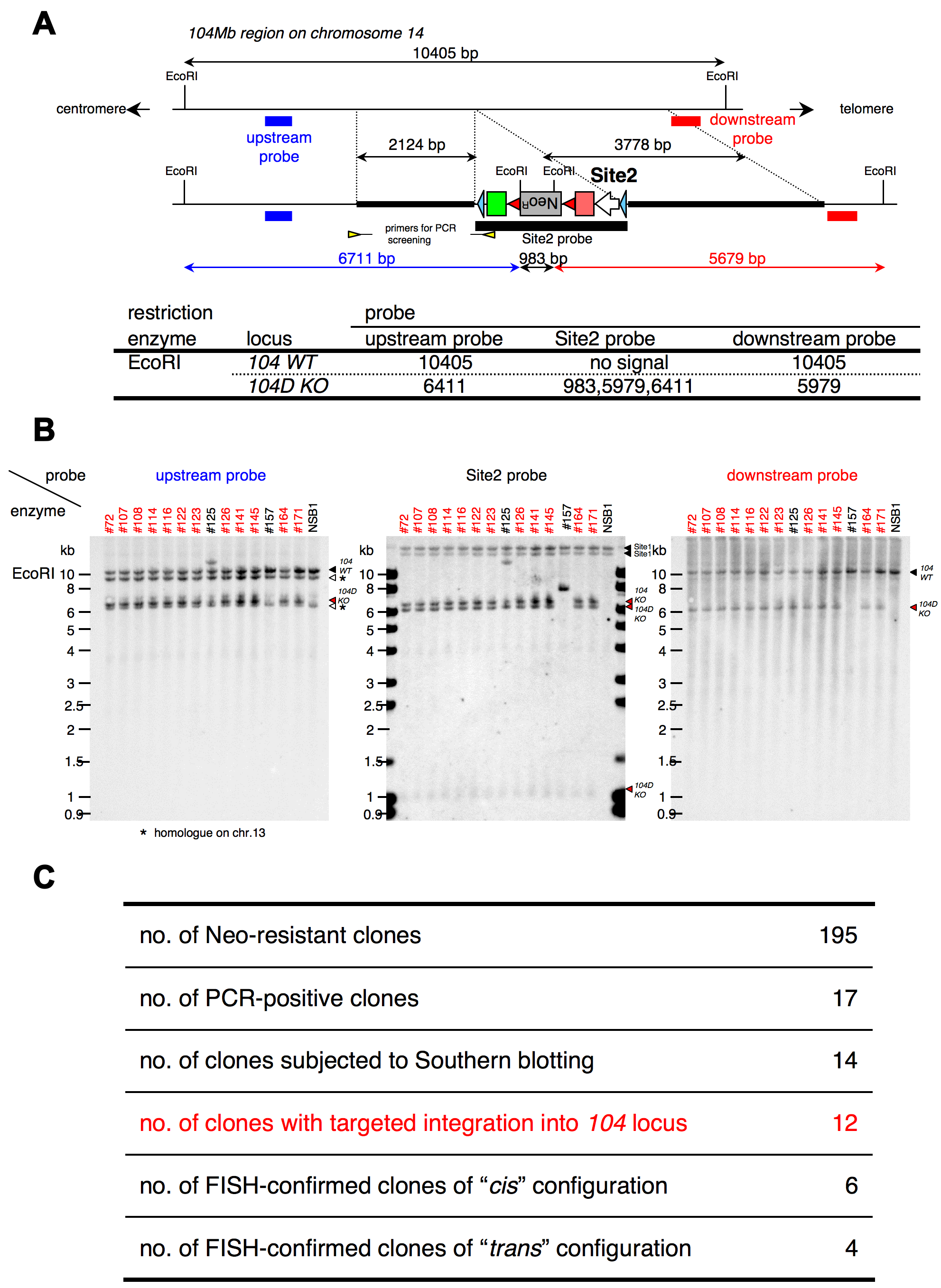

Supplement: Figure S5 — Gene targeting strategy for 104 locus for deletion/duplication and screening summary. A, EcoRI restriction map of a region at 104 Mb on the coordinate of chromosome 14, 1.1 Mb centromeric to the IGHA2 loci (upper panel), and expected band sizes on the Southern blot (table). Regions of targeting homology are indicated by parallel dotted lines with in-between numbers depicting bp. Positions of DNA probes used for Southern blotting are indicated by thick blue (upstream), black (internal) and red (downstream) lines. Numbers with bp above bidirectional arrows represent the distance between relevant restriction sites. Primer positions for PCR screening are indicated by arrowheads. Unidirectional arrows indicate direction toward the centromere or the telomere. The table lists expected sizes of bands on the Southern blot using the indicated enzymes and probes for wild-type (WT) and targeted (knockout, KO) alleles. B, Southern blot using the indicated enzymes and probes for 14 (NSK) clones and parental NSB1 cells. Outermost lanes of each panel were loaded with the size marker. Expected positions of WT and KO alleles are shown by arrowheads. Two extra bands on the upstream probe blot indicated by open arrowheads and asterisks appeared to be derived from a highly homologous sequence on chromosome 13. C, Summary of clone numbers is indicated. (1.94 MB TIF) [file pone.0009846.s007.tif]

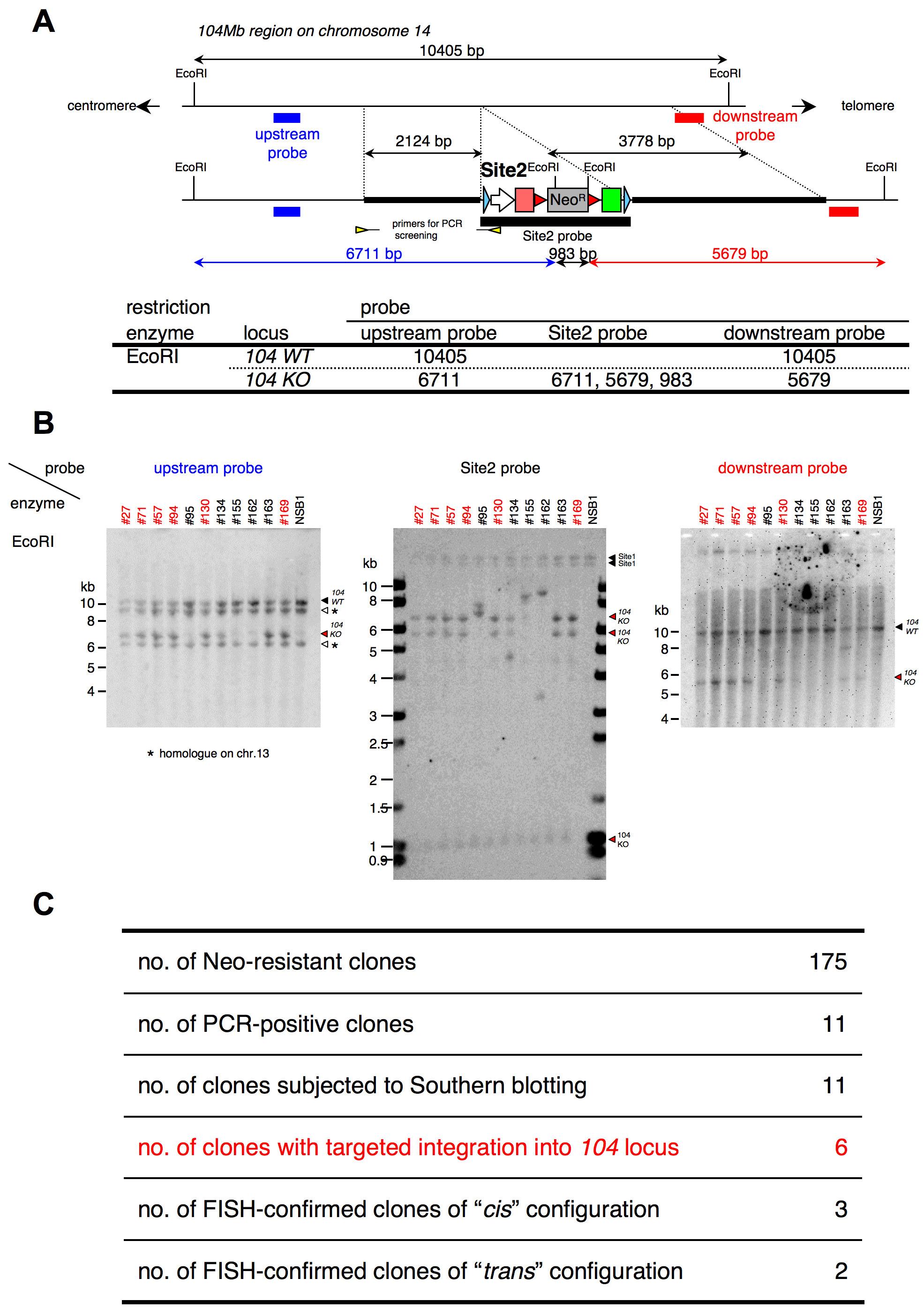

Supplement: Figure S6 — Gene targeting strategy for 104 locus for inversion and screening summary. A, EcoRI restriction map of a region at 104 Mb on the coordinate of chromosome 14, 1.1 Mb centromeric to the IGHA2 loci (upper panel), and expected band sizes on the Southern blot (table). Regions of targeting homology are indicated by parallel dotted lines with in-between numbers depicting bp. Positions of DNA probes used for Southern blotting are indicated by thick blue (upstream) and red (downstream) lines. Numbers with bp above bidirectional arrows represent the distance between relevant restriction sites. Primer positions for PCR screening are indicated by arrowheads. Unidirectional arrows indicate direction toward the centromere or the telomere. The table lists the expected sizes of bands on the Southern blotting using the indicated enzymes and probes for wild-type (WT) and targeted (knockout, KO) alleles. B, Southern blot using the indicated enzymes and probes for 11 (NSC) clones and parental NSB1 cells. Outermost lanes of each panel were loaded with the size marker. Expected positions of WT and KO alleles are shown by arrowheads. C, Summary of clone numbers is indicated. (1.86 MB TIF) [file pone.0009846.s008.tif]

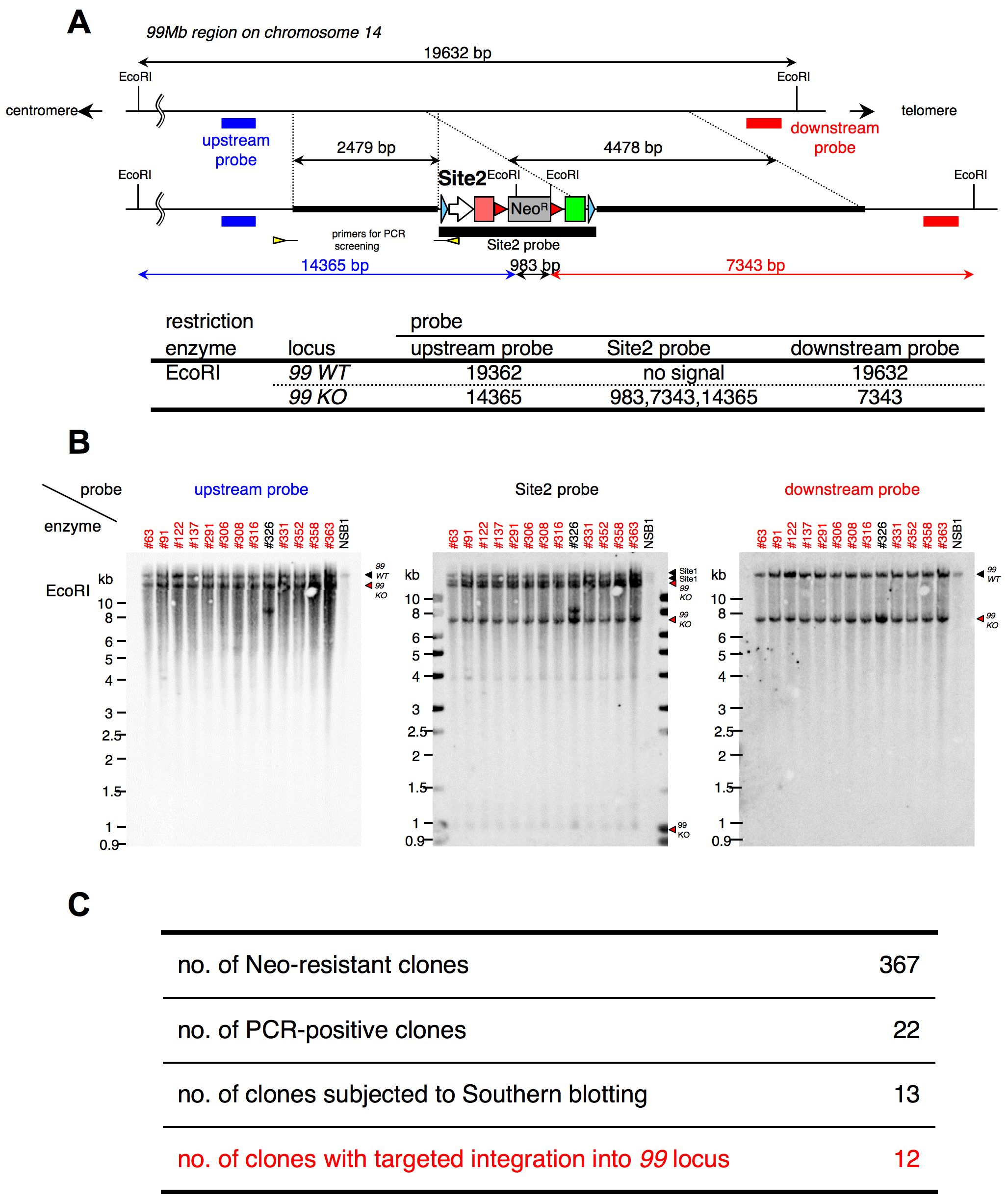

Supplement: Figure S7 — Gene targeting strategy for 99 locus for inversion and screening summary. A, EcoRI restriction map of a region at 99 Mb on the coordinate of chromosome 14, 6.1 Mb centromeric to the IGHA2 loci (upper panel), and expected band sizes on the Southern blotting (table). Regions of targeting homology are indicated by parallel dotted lines with in-between numbers depicting bp. Positions of DNA probes used for Southern blotting are indicated by thick blue (upstream), black (internal) and red (downstream) lines. Numbers with bp above bidirectional arrows represent the distance between relevant restriction sites. Primer positions for PCR screening are indicated by arrowheads. Unidirectional arrows indicate direction toward the centromere or the telomere. The table lists the expected sizes of bands on the Southern blot using the indicated enzymes and probes for wild-type (WT) and targeted (knockout, KO) alleles. B, Southern blot using the indicated enzymes and probes for 13 (NSF) clones and parental NSB1 cells. Outermost lanes of each panel were loaded with the size marker. Expected positions of WT and KO alleles are shown by arrowheads. C, Summary of clone numbers is indicated. (1.95 MB TIF) [file pone.0009846.s009.tif]

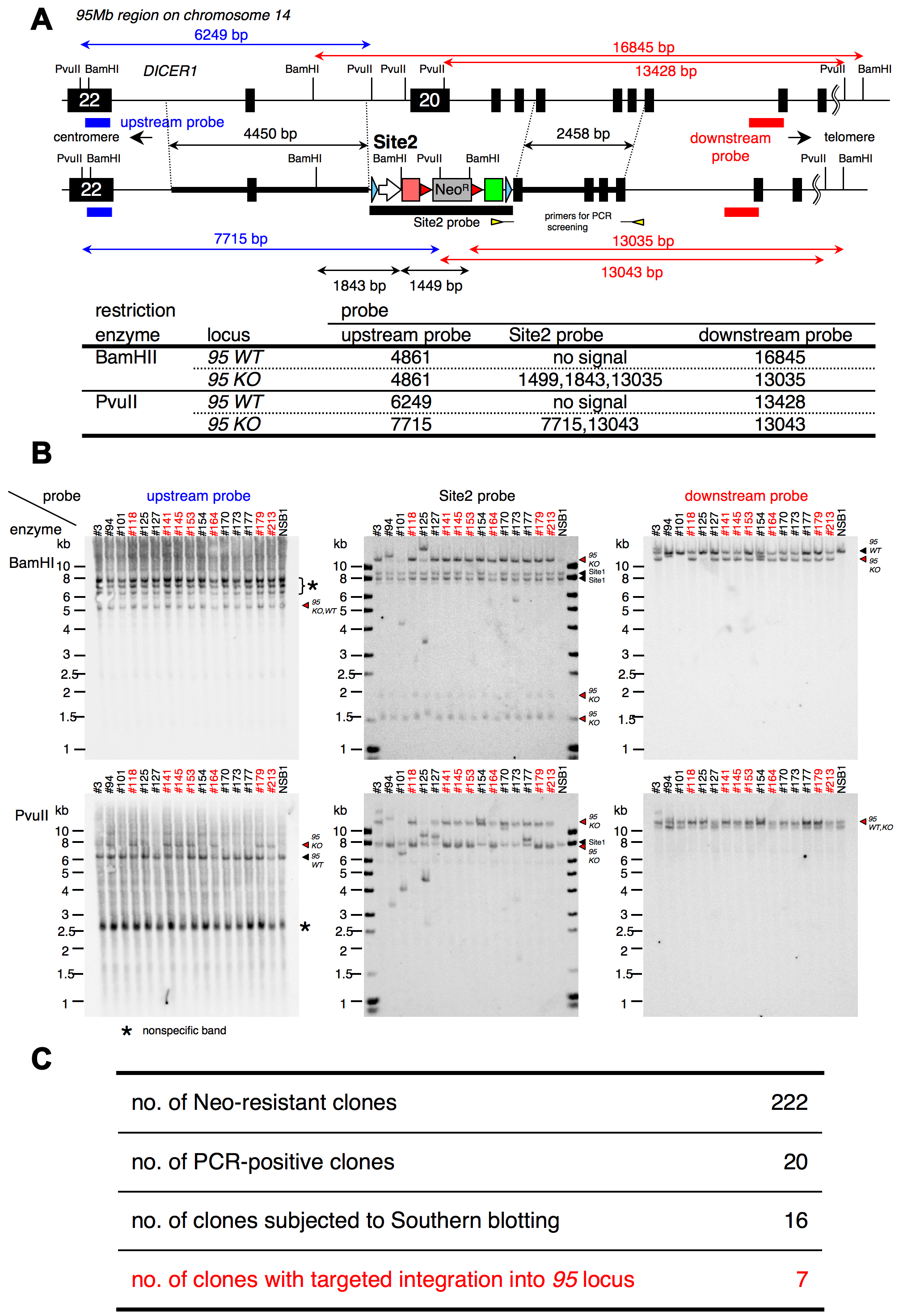

Supplement: Figure S8 — Gene targeting strategy for 95 locus for inversion and screening summary. A, Restriction map of the DICER1 locus at 95 Mb on the coordinate of chromosome 14, 10.5 Mb centromeric to the IGHA2 loci (upper panel), and expected band sizes on the Southern blot (table). Rectangles indicate exons of the DICER1 gene. Some of exon numbers are indicated in white. Regions of targeting homology are indicated by parallel dotted lines with in-between numbers depicting bp. Positions of DNA probes used for Southern blotting are indicated by thick blue (upstream), black (internal) and red (downstream) lines. Numbers with bp above bidirectional arrows represent the distance between relevant restriction sites. Primer positions for PCR screening are indicated by arrowheads. Unidirectional arrows indicate direction toward the centromere or the telomere. The table lists expected sizes of bands on the Southern blot using the indicated enzymes and probes for wild-type (WT) and targeted (knockout, KO) alleles. B, Southern blot using the indicated enzymes and probes for 16 (NSG) clones and parental NSB1 cells. Outermost lanes of each panel were loaded with the size marker. Expected positions of WT and KO alleles are shown by arrowheads. C, Summary of clone numbers is indicated. (2.94 MB TIF) [file pone.0009846.s010.tif]

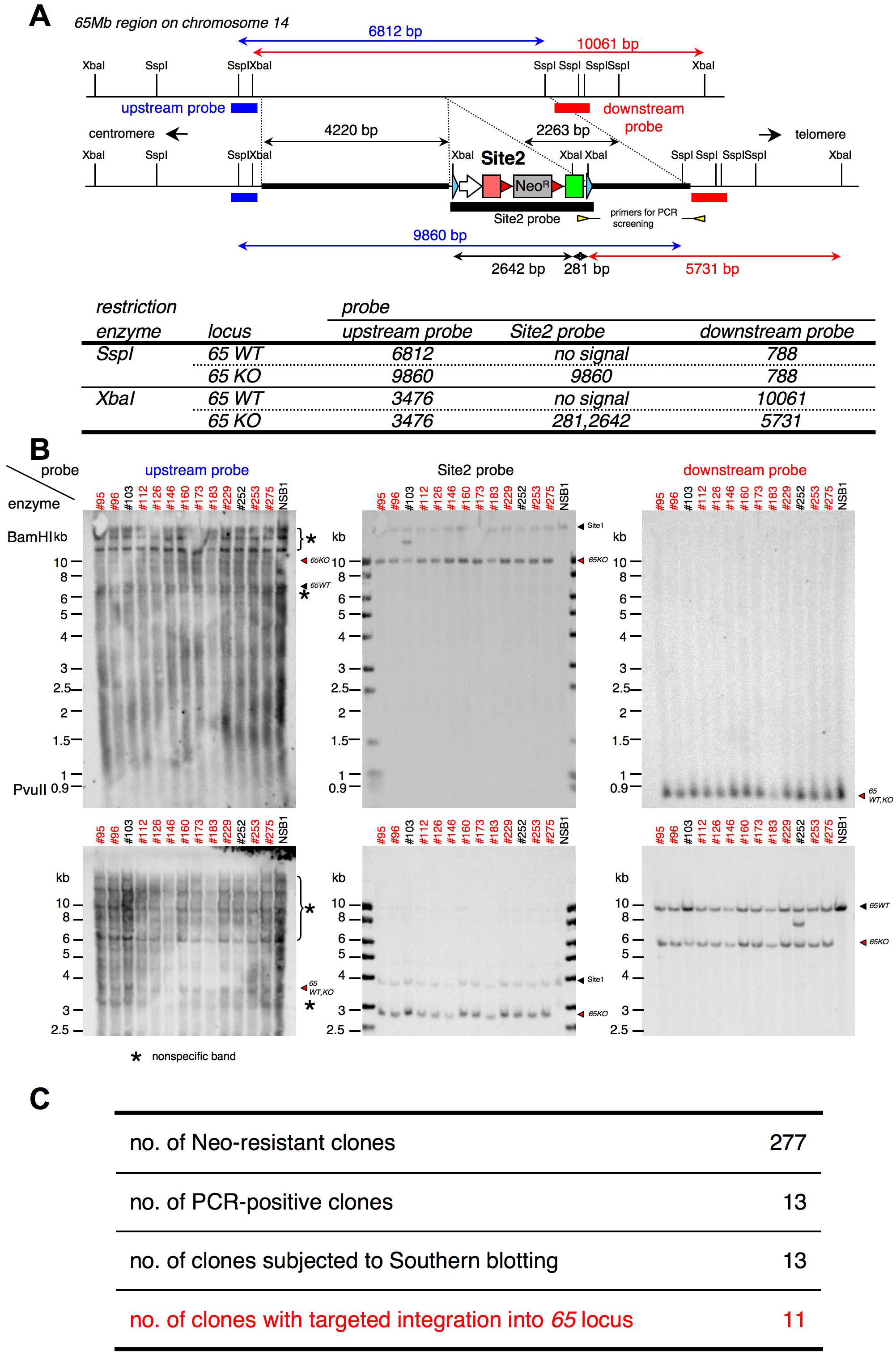

Supplement: Figure S9 — Gene targeting strategy for 65 locus for inversion and screening summary. A, Restriction map of a region at 65 Mb on the coordinate of chromosome 14, 39.8 Mb centromeric to the IGHA2 loci (upper panel), and expected band sizes on the Southern blot (table). Regions of targeting homology are indicated by parallel dotted lines with in-between numbers depicting bp. Positions of DNA probes used for Southern blotting are indicated by thick blue (upstream), black (internal) and red (downstream) lines. Numbers with bp above bidirectional arrows represent the distance between relevant restriction sites. Primer positions for PCR screening are indicated by arrowheads. Unidirectional arrows indicate direction toward the centromere or the telomere. The table lists the expected sizes of bands on the Southern blot using the indicated enzymes and probes for wild-type (WT) and targeted (knockout, KO) alleles. B, Southern blot using the indicated enzymes and probes for 13 (NSI) clones and parental NSB1 cells. Outermost lanes of each panel were loaded with the size marker. Expected positions of WT and KO alleles are shown by arrowheads. C, Summary of clone numbers is indicated. (3.14 MB TIF) [file pone.0009846.s011.tif]

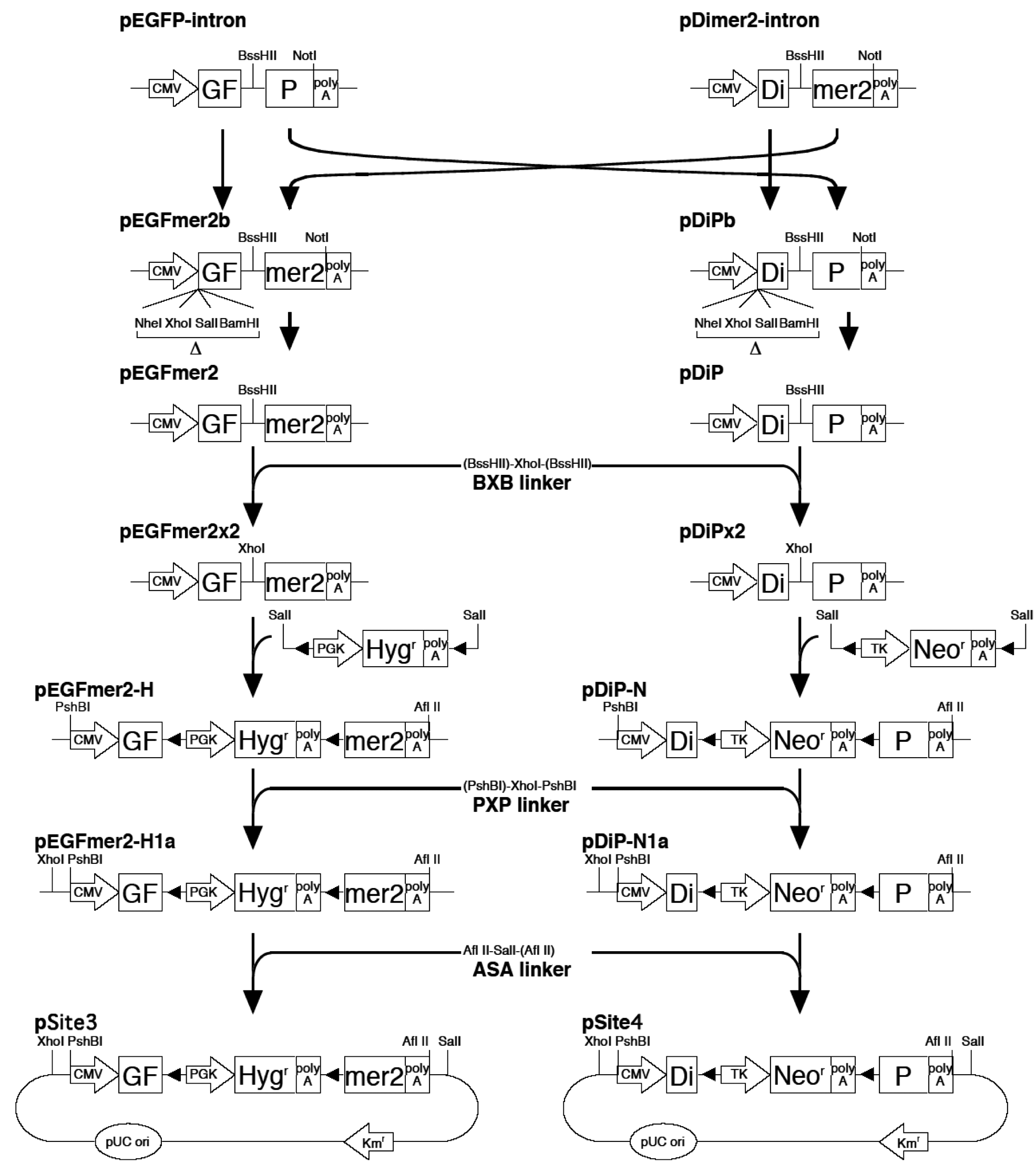

Supplement: Figure S10 — Construction scheme part 1. For details, see Methods S1. (0.61 MB TIF) [file pone.0009846.s012.tif]

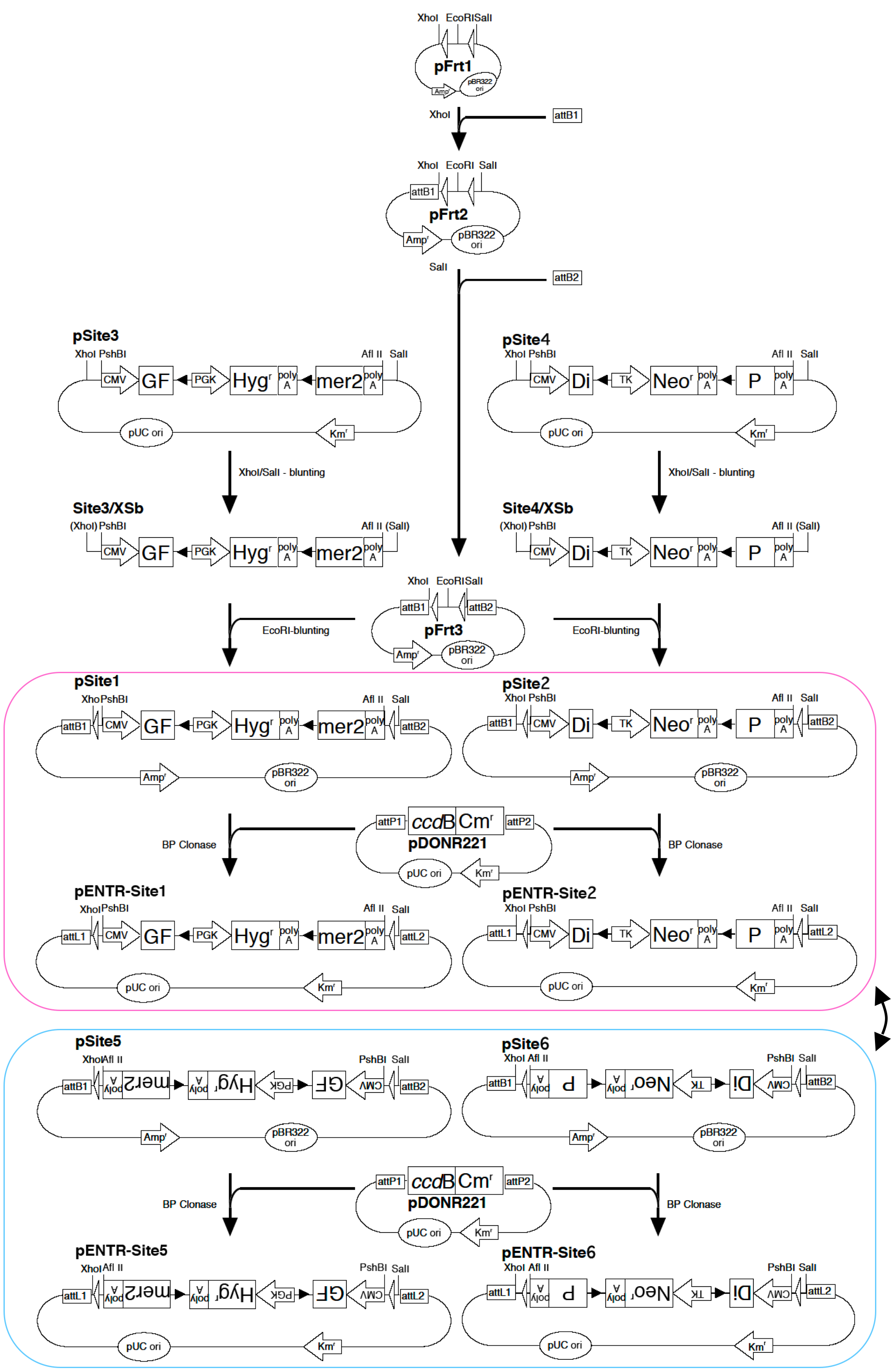

Supplement: Figure S11 — Construction scheme part 2. For details, see Methods S1. (1.74 MB TIF) [file pone.0009846.s013.tif]
